# Safety and Immunomodulatory Effects of Three Probiotic Strains Isolated from the Feces of Breast-Fed Infants in Healthy Adults: SETOPROB Study

**DOI:** 10.1371/journal.pone.0078111

**Published:** 2013-10-28

**Authors:** Julio Plaza-Diaz, Carolina Gomez-Llorente, Laura Campaña-Martin, Esther Matencio, Inmaculada Ortuño, Rosario Martínez-Silla, Carlos Gomez-Gallego, Maria Jesús Periago, Gaspar Ros, Empar Chenoll, Salvador Genovés, Beatriz Casinos, Ángela Silva, Dolores Corella, Olga Portolés, Fernando Romero, Daniel Ramón, Antonio Perez de la Cruz, Angel Gil, Luis Fontana

**Affiliations:** 1 Department of Biochemistry & Molecular Biology II, School of Pharmacy, University of Granada, Granada, Spain; 2 Institute of Nutrition & Food Technology “José Mataix”, Biomedical Research Center, University of Granada, Granada, Spain; 3 Hero Global Technology Center, Hero Spain, S.A., Alcantarilla, Murcia, Spain; 4 Department of Human Nutrition and Food Science, Faculty of Veterinary Sciences, University of Murcia, Murcia, Spain; 5 Department of Food Biotechnology, Biopolis s.l., Parc Científic Universitat de Valencia, Paterna, Valencia, Spain; 6 Department of Preventive Medicine and Public Health, School of Medicine, University of Valencia, Valencia, Spain; 7 CIBER Fisiopatologia de la Obesidad y Nutricion, Instituto de Salud Carlos III, Madrid, Spain; 8 Unit of Nutrition and Dietetics, Virgen de las Nieves Hospital, Granada, Spain; University of Pittsburgh, United States of America

## Abstract

We previously described the isolation and characterization of three probiotic strains from the feces of exclusively breast-fed newborn infants: *Lactobacillus paracasei* CNCM I-4034, *Bifidobacterium breve* CNCM I-4035 and *Lactobacillus rhamnosus* CNCM I-4036. These strains were shown to adhere to intestinal mucus *in vitro*, to be sensitive to antibiotics and to resist biliary salts and low pH. In the present study, a multicenter, randomized, double-blind, placebo-controlled trial with 100 healthy volunteers in three Spanish cities was carried out to evaluate the tolerance, safety, gut colonization and immunomodulatory effects of these three probiotics. Volunteers underwent a 15-day washout period, after which they were randomly divided into 5 groups that received daily a placebo, a capsule containing one of the 3 strains or a capsule containing a mixture of two strains for 30 days. The intervention was followed by another 15-day washout period. Patients did not consume fermented milk for the entire duration of the study. Gastrointestinal symptoms, defecation frequency and stool consistency were not altered by probiotic intake. No relevant changes in blood and serum, as well as no adverse events occurred during or after treatment. Probiotic administration slightly modified bacterial populations in the volunteers’ feces. Intestinal persistence occurred in volunteers who received *L. rhamnosus* CNCM I-4036. Administration of *B. breve* CNCM I-4035 resulted in a significant increase in fecal secretory IgA content. IL-4 and IL-10 increased, whereas IL-12 decreased in the serum of volunteers treated with any of the three strains. These results demonstrate that the consumption of these three bacterial strains was safe and exerted varying degrees of immunomodulatory effects.

**Trial Registration:**

ClinicalTrials.gov NCT01479543

## Introduction

The Food and Agriculture Organization (FAO) and the World Health Organization (WHO) deﬁne probiotics as live microorganisms that confer a health beneﬁt to the host when administered in adequate amounts [1]. Strains belonging to *Bifidobacterium* and *Lactobacillus*, the predominant and subdominant groups of the gastrointestinal microbiota, respectively [2,3], are the most widely used probiotic bacteria and are included in many functional foods and dietary supplements [4-6]. 

The FAO/WHO [1] and the European Union (EU)-funded Product Safety Enforcement Forum of Europe (EU-PROSAFE) project [7] have attempted to create consensus guidelines for probiotic safety evaluation. These groups have recommended that i) the genus and species of the microorganism must ﬁrst be deﬁnitively determined by phenotypic and genotypic techniques, ii) the strains must be deposited in an internationally recognized culture collection, and iii) the safety of the bacterial strain must be evaluated through acute ingestion studies in murine models and the estimation of potential side effects in human studies.

For probiotics to be successful, they must possess certain characteristics. The criteria for the selection of probiotics include tolerance to gastrointestinal conditions (gastric acid and bile), ability to adhere to the gastrointestinal mucosa and competitive exclusion of pathogens [8,9]. 

We have previously described the isolation of three lactic acid bacteria (LAB) strains from the feces of exclusively breast-fed newborn infants. These strains were selected based on their probiotic properties, such as adhesion to intestinal mucus, sensitivity to antibiotics and resistance to biliary salts and low pH. We identified these strains as *Lactobacillus paracasei* CNCM I-4034, *Bifidobacterium breve* CNCM I-4035 and *Lactobacillus rhamnosus* CNCM I-4036 [10]. In addition, their safety has been assessed by acute ingestion in immunocompetent and immunosuppressed BALB/c mouse models. The three strains inhibited *Listeria monocytogenes*, the etiological agent of meningitis, and human rotavirus infections *in vitro* [10].

The immunomodulatory effects of probiotics have been demonstrated in experimental models of allergy, autoimmunity and inﬂammatory bowel disease [2]. In the present study, a multicentric, randomized, double-blind placebo-controlled trial with healthy volunteers was undertaken to investigate the tolerance, safety and colonization of the aforementioned probiotic strains, following the FAO/WHO guidelines [1]. Additionally, we have evaluated their potential immunomodulatory effects by quantitating cytokines and secretory IgA in volunteers’ serum and feces, respectively.

## Materials and Methods

### Ethical statement

All patients enrolled in this study signed an informed consent form. The study followed the guidelines laid down in the Declaration of Helsinki and was approved by the ethics review committees of the University of Granada, Murcia and Valencia.

### Probiotics

The probiotic strains *Lactobacillus paracasei* CNCM I-4034, *Bifidobacterium breve* CNCM I-4035 and *Lactobacillus rhamnosus* CNCM I-4036 have been described elsewhere [10]. These strains were assayed for enzymatic activity and carbohydrate utilization, and they were deposited in the Collection Nationale de Cultures de Microorganismes (CNCM) of the Institute Pasteur [10].

### Experimental design

**Figure 1:**
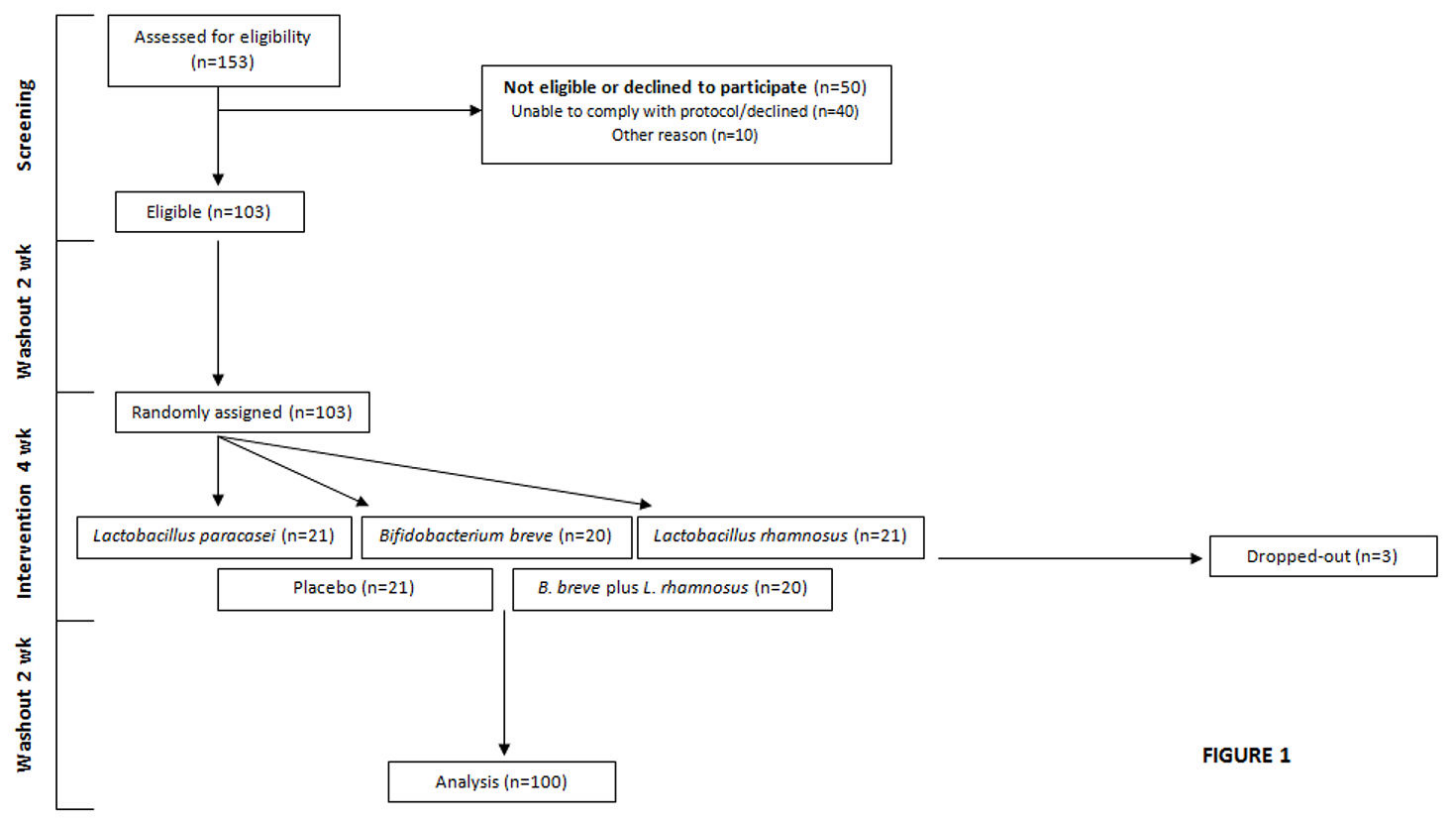
CONSORT flow diagram of the subjects in the SETOPROB study (NCT01479543).

This study was a multicenter, randomized, double-blind, placebo-controlled trial. The trial was registered at www.clinicaltrials.gov as NCT01479543. Randomization was simple and not subjected to any kind of restriction such as blocking or block size. One hundred and three healthy volunteers were enrolled in three different cities in Spain (Granada, Murcia and Valencia). We used a random allocation sequence [11]. Briefly, each city was assigned 35 codes taken from a randomization table. Six codes were assigned to each treatment in each city. Envelopes containing the codes and matching those in the randomization table were assigned to each participant. Volunteers were enrolled and assigned by Gomez-Llorente C (Granada), Ros G (Murcia) and Corella D (Valencia). This was a double blind study. A flow chart of the study design is depicted in Figure 1. The protocol for this trial and supporting CONSORT checklist are available as supporting information; see Checklist S1 and Protocol S1.

Volunteers underwent a 15-day washout period (t_1_), after which they were randomly and blindly divided into 5 groups that received daily either a placebo, a capsule containing 9x10^9^ CFUs of one of the 3 strains, or a capsule containing 9x10^9^ CFUs of a mixture of *B. breve* CNCM I-4035 and *L. rhamnosus* CNCM I-4036, for 30 days (t_2_).

The placebo contained 67% cow’s milk powder, 32.5% sucrose, and 0.56% vitamin C. The 30-day intervention period was followed by a second washout of another 15 days (t_3_) (Figure 1). Patients did not consume any fermented milk for the entire duration of the study. Blood samples were taken at t_1_ and t_2_. Blood was centrifuged to separate serum from cells. Fecal samples were taken at t_1_, t_2_ and t_3_. Baseline data appear in Table 1.

**Table 1:** Baseline characteristics of the study groups.

	Probiotic groups	Placebo group
	(n=80)	(n=20)
Sex (male/female)	37/43	9/11
Age (years)	28.7 ± 0.7	28.5 ± 1.7
Height (m)	1.71 ± 0.1	1.7 ± 0.1
Weight (kg)	68.4 ± 1.4	67 ± 2.5
BMI (kg/m^2^)	23.1 ± 0.4	22.8 ± 0.5
Heart rate (beats/min)	72.0 ± 1.5	71.2 ± 3.0
Blood pressure (mm/Hg)		
Systolic	116.8 ± 1.8	117.6 ± 3.1
Diastolic	71.5 ± 1.2	71.6 ± 2.3

Values are means ± SEM unless otherwise indicated. There were no significant differences between groups.

Volunteers were recruited between July and October 2011. The first washout was in October 2011. Intervention ended in November 2011, and the second washout in December 2011. All determinations were finished by December 2012.

Primary outcome variables were safety, tolerance and persistence. Secondary outcome variables were bacterial populations, immunomodulatory effects (cytokine and secretory IgA production), microbiological analyses, and lymphocyte populations. Calculation of sample size was done based on the variance in the main outcome variable persistence, i.e., probiotic strain count (log strain CFU/g) in feces and a difference of 25% compared with the placebo (12). A type 1 error of α=0.05 and a power of 90%, (β=0.1) were assumed. The calculated minimum number of subjects per group was 19. The initial number of volunteers per group was as follows: placebo, n=21; *L. paracasei* CNCM I-4034, n=21; *B. breve* CNCM I-4035, n=20; *L. rhamnosus* CNCM I-4036, n=21; mixture of *B. breve* CNCM I-4035 and *L. rhamnosus* CNCM I-4036, n=20. One subject each of groups placebo, *L. paracasei* CNCM I-4034 and *L. rhamnosus* CNCM I-4036 voluntarily dropped out of the study. No changes in the estimated sample size or its precision occurred as all selected volunteers received intended treatment and were analyzed.

The *inclusion criteria* were as follows: healthy male or female, age 18–50 years, normal defecation pattern, blood parameters within the normal range or not considered clinically significant if outside of the normal range, BMI 18.0–29.9 kg/m^2^ and written informed consent. The *exclusion criteria* were pregnancy or breast-feeding, blood parameters outside of the normal range and considered clinically significant, a history of metabolic or gastrointestinal disease, food allergies, recent use of antibiotics or laxative drugs, diarrhea, constipation, diabetes mellitus, smoking and blood pressure > 140/90 mmHg.

Determinations described below were carried out in all volunteers (20 per group) with the exception of antibiotic resistance, which was done in Valencia (3 volunteers/group, n=15).

### Collection and preparation of fecal samples

Fecal samples were collected from each volunteer in plastic pots lined with a sterile plastic bag in anaerobic conditions and submitted immediately by courier to the laboratory. Samples were analyzed within a maximum of 4 h. 

### Gastrointestinal tolerance and safety parameters

Gastrointestinal tolerance was determined using the gastrointestinal symptom rating scale (GSRS) [13], the King’s Stool Chart for stool consistency [14], daily recorded gastrointestinal symptoms (nausea, vomiting, diarrhea, burping, abdominal distension and flatulence) [15] and defecation frequency. Baseline GSRS and stool consistency were measured by the investigator and at 4 and 6 weeks. Product compliance was recorded daily in a diary. Intolerance was defined as a symptom score of 2 or higher (moderate or severe) on the GSRS. Safety parameters were the number and type of adverse events recorded throughout the entire study and changes from baseline blood parameters determined at the end of the supplementation period. The measurement of blood parameters was performed in the Clinical Analysis Laboratory of the Virgen de las Nieves Hospital (Granada), Clinical Hospital (Valencia) and Megalab laboratory (Murcia).

### Fluorescence *in situ* hybridization-flow cytometry analysis (FISH-FC)

Fecal samples were processed as previously described [16,17]. One gram of feces was homogenized in 9 mL of PBS (phosphate-buffered saline), and then 0.2 mL of the suspension was mixed with 0.6 mL of 4% paraformaldehyde (PFA) in PBS and fixed overnight at 4°C. Fecal bacterial populations were assessed by FISH-CF analysis as described by Fallani et al. and Gomez-Llorente et al. [16,17]. A panel of 10 group- and species-specific probes covalently linked with Cy5 at their 5’ end was used to assess the microbiota composition [18-25] (Table S1). 

Hybridization was performed in a 96-well microtiter plate overnight at 35°C in hybridization solution containing 4 ng/µL of the appropriate probes, and then 150 µL of hybridization solution was added to each well. Cells were pelleted and washed to remove any nonspecific probe binding by incubating the bacterial cells at 37°C for 20 min in wash solution (64 mmol/L NaCl, 20 mmol/L Tris-HCl, pH 8.0, 5 mmol/L EDTA pH 8.0, 0.01% sodium dodecyl sulfate, pH 7.2). Finally, the cells were pelleted and resuspended in PBS. Samples were analyzed in a FACSCanto II flow cytometer (Becton Dickinson, NJ, USA). 

### Microbiological analysis

Fecal samples were analyzed by plating appropriate dilutions onto Wilkins-Chalgren agar (Panreac Quimica, Barcelona, Spain) to determine the total number of anaerobic bacteria, de Man-Rogosa-Sharpe (MRS) agar (Oxoid, Basingstoke, United Kingdom) to determine the number of *Lactobacilli*, and Beerens agar (Oxoid, Basingstoke, United Kingdom) to determine the number of bifidobacteria. The *Lactobacillus rhamnosus* CNCM I-4036 count was determined on modified MRS medium in which glucose was substituted with rhamnose. Only *L. rhamnosus* and a few other rare *Lactobacillus* species in the human gastrointestinal tract are able to grow on this medium [12,26].

### Antibiotic resistance analysis

The sensitivity of probiotic strains to ampicillin and tetracycline was analyzed in volunteers’ fecal samples by plating appropriate dilutions onto MRS agar (Oxoid, Basingstoke, United Kingdom) supplemented with 0.05% (wt/vol) cysteine (Sigma-Aldrich) (MRS-C medium) and trypticase soy agar (TSA, Oxoid) with or without ampicillin (2 and 4 µg/mL; Sigma-Aldrich, St. Louis, MO) or tetracycline (4 and 8 µg/mL; Sigma-Aldrich). The plates were incubated for 48-72 h at 37°C in an anaerobic atmosphere, which was generated using an AnaeroGen® system, for MRS and MRS-C, and aerobically at 30°C in the case of TSA plates. 

### Isolation procedure

From each patient, 5 to 10 random colonies grown on modified MRS agar were individually inoculated into MRS broth medium (Oxoid, Basingstoke, United Kingdom) for 2 days, at 37°C under anaerobic conditions (Anaerogen® Oxoid, Basingstoke, United Kingdom). DNA was extracted with the QIAamp DNA Mini Kit (QIAGEN, Barcelona, Spain) and used for identification by quantitative real-time PCR (qPCR) with specific primers (see Table S2) for this probiotic strain.

### Real-time polymerase chain reaction (PCR)

Real-time PCR was used i) to identify *Lactobacillus*, *Bifidobacterium* spp., *Bacteroides* and *Clostridium difficile* in feces and ii) to confirm intestinal persistence by *Lactobacillus rhamnosus*. For the former (i), DNA was isolated from volunteers’ feces with the QIAamp DNA Stool Mini Kit (QIAGEN, Barcelona, Spain). For the latter (ii), DNA was isolated from bacterial cultures (see sections Microbiological determinations and Re-isolation procedure). The primer sequences appear in Table S2.

PCR was performed in triplicate in an Eppendorf Mastercycle EP Gradient. The primer sequences are shown in Table S2 and were purchased from Sigma-Aldrich (Barcelona, Spain). PCR was carried out using Power SYBR Green Master Mix (Applied Biosystems, Barcelona, Spain). The PCR program was as follows: an initial activation/denaturation step at 95°C for 5 min followed by 30-40 cycles of 15 sec at 95°C, 30-40 sec for annealing at 55-68°C and a final extension step for 33-45 s at 72°C. Quantitation was performed using a standard curve.

In the case of strain-specific reactions, PCR was performed in triplicate in the StepOne Real-Time PCR System (ABI). Primers were purchased from Thermo Fisher (Thermo Fisher Scientific, Waltham, MA). PCR was carried out using Power SYBR Green Master Mix (ABI). Taq polymerase was activated at 95°C for 10 min. The cycling parameters were denaturation at 95°C for 15 sec and extension at 64°C for 50 sec (for 30 cycles). Colonies were identified as *L. rhamnosus* CNCM I-4036 when amplification appeared. 

### Determination of the fecal content of secretory IgA

Secretory IgA was analyzed in feces by enzyme-linked immunosorbant assay (Immundiagnostik AG, Bensheim, Germany) according to the manufacturer’s instructions.

### Preparation and collection of blood samples

Blood samples were collected into BD Vacutainer® tubes (Becton Dickinson, NJ, USA). An aliquot of the blood was used for hematological determination. A second aliquot was centrifuged for 10 min at 1000 x g and 4°C to separate serum from cells. Serum was collected for cytokine analysis. 

### Determination of differences in the lymphocyte population by fluorescence-activated cell sorting (FACS)

These analyses were performed at the University of Murcia in the 24-hour period after blood collection to avoid cell lysis. PerCP-Cy-conjugated anti-human CD14, PE-conjugated anti-CD4, FITC-conjugated anti-CD4, PE-Cy7 conjugated anti-CD25, AlexaFluor®-conjugated anti-CD127, PE-conjugated anti-CD19 and PerCP-conjugated anti-CD8 antibodies purchased from Becton Dickinson (San Diego, California, USA) were used to perform multicolor flow cytometric analysis.

The monoclonal antibodies were incubated with 200 µL of the whole blood samples obtained from volunteers for 15 min protected from light. Erythrocytes were removed by hypotonic lysis using Pharm Lyse^TM^ (BD Biosciences, San Diego, CA), and samples were cleaned according to the manufacturer’s instructions. Flow cytometry was performed using a fluorescence activated cell sorter (FACS) Calibur® flow cytometer (Becton Dickinson) and Cell Quest (BD). For each antibody panel analysis, 2х10^4^ lymphocytes were gated.

### Cytokine quantification in serum

IL-4, IL-6, IL-10, IL-12(p70), TNF-α, and TGF-β were measured using MILLIplex^TM^ immunoassays (Merck-Millipore, MA, USA) on the Luminex 200 system according to the manufacturer’s instructions.

### Statistical analysis

All results are expressed as the mean ± SEM unless otherwise indicated. Statistical analyses of gastrointestinal symptom scores were performed using the Mann–Whitney *U* test for equivalence. Time comparisons for normally distributed parameters were tested for statistical significance by a lineal model of variance for repeated measures. For those variables found significantly different, specific time differences were tested using the paired t test while the paired Wilcoxon test was used for non-normally distributed parameters. All analyses were performed using the statistical package IBM SPSS (Statistical Package for the Social Sciences) Statistics 20 (Somers, NY, USA).

## Results

### Subjects

Of the 103 patients enrolled in the study, 3 dropped out during the intervention period (Figure 1). Baseline features of the volunteers appear in Table 1. The average age was 28 years in the placebo and probiotic groups. There was no significant difference between volunteers who received placebo and those fed probiotics regarding height, weight, body mass index, heart rate or blood pressure at baseline.

### Tolerance and safety

Symptom scores as measured by the GSRS questionnaire are described in Table 2. All symptom scores were less than 2, and there was no significant difference between the control group and the probiotic-treated group. The median score of the daily recorded gastrointestinal symptoms of acid regurgitation, nausea, vomiting, abdominal distension, and eructation did not change during the probiotic supplementation (intervention) and subsequent follow-up period. Additionally, the stool consistency and defecation frequency did not change during the supplementation period and the subsequent follow-up period in the probiotic and placebo groups (Table S3) 

**Table 2:** Gastrointestinal symptom score according to the Gastrointestinal Symptom Rating Scale (GSRS).

						GSRS symptom score						
			Probiotic groups (n=80)						Placebo group (n=20)			
Symptom	t_1_		t_2_		t_3_		t_1_		t_2_		t_3_	
	Median	Range	Median	Range	Median	Range	Median	Range	Median	Range	Median	Range
Abdominal pain (q1)	0		0	0-1	0		0		0		0	
Heartburn (q2)	0		0	0-1	0	0-1	0	0-1	0	0-1	0	
Acid regurgitation (q3)	0	0-1	0	0-1	0	0-1	0		0	0-1	0	0-1
Sucking sensations in the epigastrium (q4)	0		0	0-1	0	0-1	0	0-1	0	0-1	0	0-1
Nausea and vomiting (q5)	0	0-1	0	0-1	0	0-1	0	0-1	0		0	
Borborygmus (q6)	0		0	0-1	0		0		0		0	
Abdominal distension (q7)	0	0-1	0	0-1	0	0-1	0	0-1	0	0-2	0	0-1
Eructation (q8)	0	0-1	0	0-1	0	0-1	0	0-1	0	0-2	0	0-1
Loose stools (q12)	0	0-2	0	0-2	0	0-1	0	0-1	0	0-2	0	0-1
Hard stools (q13)	0	0-2	0	0-2	0	0-1	0	0-1	0	0-2	0	0-1
Urgent need for defecation (q14)	0	0-1	0	0-1	0		0	0-1	0	0-1	0	0-1
Sensation of incomplete evacuation (q15)	0		0		0		0		0		0	
Dyspeptic syndrome (q1-5)	0	0-0.4	0	0-0.8	0	0-0.8	0	0-0.2	0	0.6	0	0.4
Indigestion syndrome (q6-8)	0	0-0.67	0	0-1	0	0-0.67	0	0-0.8	0	0-1	0	0-0.8
Bowel dysfunction syndrome (q12-15)	0	0-1	0	0-1	0	0-0.67	0	0-1	0	0-1	0	0-1

Values are the median and range. q, question number of questionnaire. 0 = absent; 1 = mild; 2 = moderate; 3 = severe. t_1_, first washout; t_2_, intervention; t_3 ,_ second washout.

Therefore, no serious adverse events occurred during the supplementation period in any of the groups based on the GSRS questionnaire, which shows that the differences between the probiotic and placebo groups were not significant for any of the reported symptoms.

Likewise, no difference between placebo and probiotic groups occurred in any of the hematological (hemoglobin, hematocrit, mean corpuscular volume, and leucocyte count) and biochemical (cholesterol, glucose, AST, ALT, γ-GT, and creatinine) parameters (Table 3). There was an initial significant difference in γ-GT between placebo and probiotics (17.6 ± 1.2 vs. 14.0 ± 0.9 probiotics vs. placebo at t_1_. *P*=0.021); however, such difference in γ-GT remained after the intervention (17.0 ± 1.1 vs. 13.4 ± 0.8 probiotics vs. placebo at t_2_. *P*=0.011).

**Table 3:** Volunteers’ hematological and biochemical data.

Parameter	Probiotic groups (n=80)		Placebo group (n=20)	
	t_1_	t_2_	t_1_	t_2_
Hemoglobin	14.2 ± 0.2	14.1 ± 0.2	14.0 ± 0.3	13.9 ± 0.3
Hematocrit	42.7 ± 0.8	41.4 ± 0.5	41.7 ± 0.9	41.2 ± 1
Mean Corpuscular Volume	88.2 ± 0.5	87.7 ± 0.5	86.5 ± 1.2	86.0 ± 1.1
Leucocytes	6.2 ± 0.1	6.0 ± 0.2	6.4 ± 0.3	6.2 ± 0.4
Total cholesterol	182.4 ± 3.8	182.3 ± 4	180.0 ± 4.6	183.0 ± 7.5
HDL-cholesterol	65.6 ± 1.9	65.0 ± 2	59.0 ± 3	58.0 ± 3.7
LDL-cholesterol	103.1 ± 3.2	104.8 ± 3.6	109.0 ± 3.4	112.0 ± 6.1
Glucose	83.9 ± 0.9	83.4 ± 0.4	85.0 ± 1.8	86.4 ± 1.8
Aspartate transaminase	23.7 ± 1.5	22.3 ± 1.0	25.4 ± 2.1	23.9 ± 1.5
Alanine transaminase	19.1 ± 1.3	21.5 ± 1.2	18.8 ± 1.9	21.1 ± 1.7
γ-glutamyl transferase	17.6 ± 1.2^*^	17.0 ± 1.1^*^	14.0 ± 0.9	13.4 ± 0.8
Creatinine	0.8 ± 0.01	0.8 ± 0.02	0.8 ± 0.03	0.8 ± 0.03

Values are means ± SEM. Hemoglobin (g/dL), hematocrit (%), mean corpuscular volume (fL), leukocytes (x10^3^/µL), total cholesterol (mg/dL), HDL-cholesterol (mg/dL), LDL-cholesterol (mg/dL), glucose (mg/dL), aspartate transaminase (U/L), alanine transaminase (U/L), γ-glutamyl transferase (U/L), creatinine (mg/dL). *P<0.05 probiotic groups vs. placebo; t_1_, first washout; t_2_, intervention.

All three probiotic strains were found to be sensitive to ampicillin and tetracycline. In addition, antibiotic sensitivity was similar among the strains at both t_1_ and at t_2_ (Table S4).

Overall, these results indicate that the three probiotic strains were safe and well tolerated by healthy subjects. 

### Fecal bacterial populations

**Figure 2:**
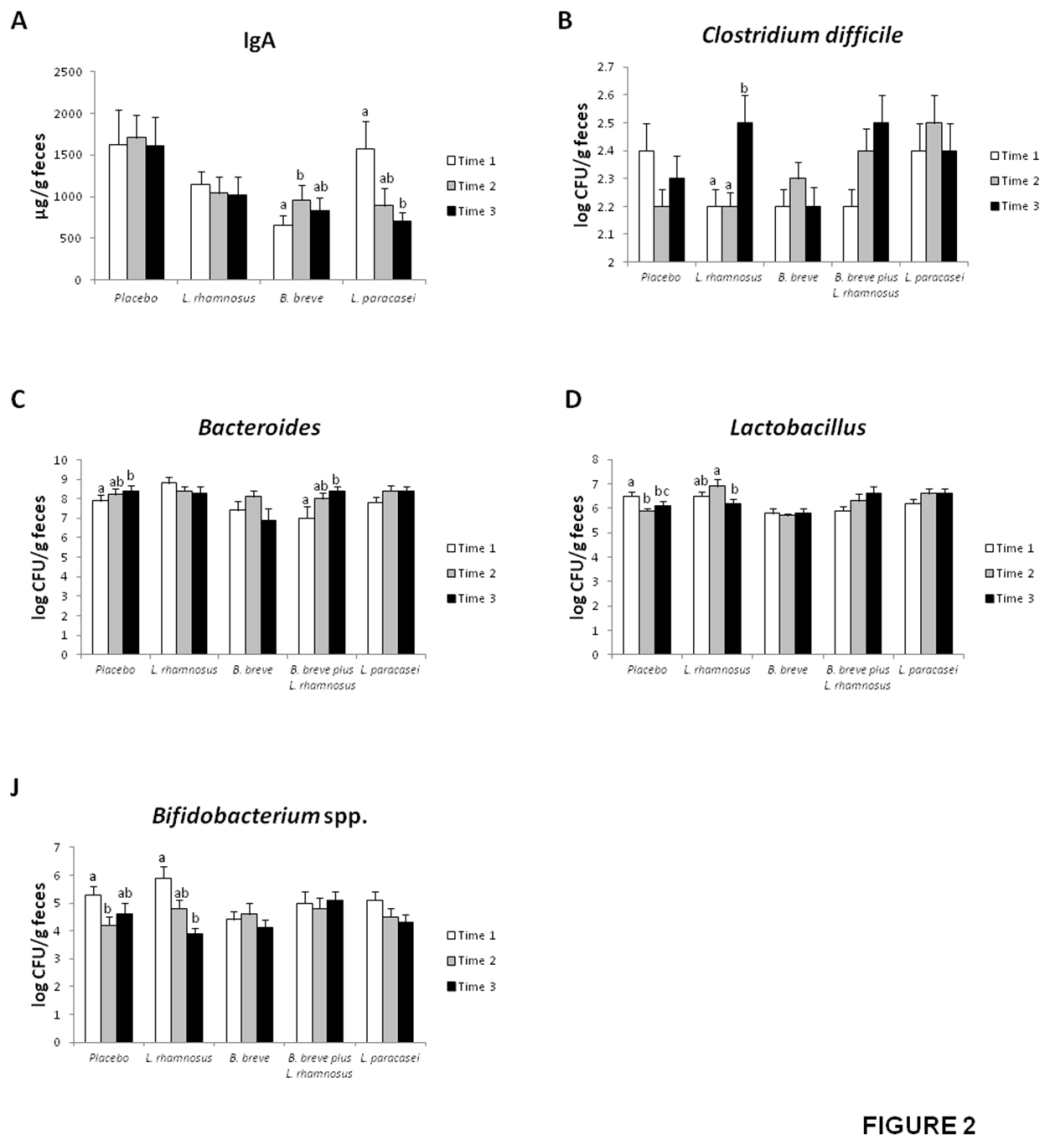
Secretory IgA content (A) and populations of *Clostridium difficile* (B), *Bacteroides* (C), *Lactobacillus* (D), and *Bifidobacterium* spp. (E) in the feces of healthy adults fed one daily probiotic capsule or placebo for 4 weeks as log CFU/g feces. Values are means ± SEM, n=20 per group. Labeled means without a common letter differ, *P*<0.05. Time 1, first washout; Time 2, intervention; Time 3, second washout.

FISH and real-time PCR were used to investigate whether fecal bacterial populations changed due to the various treatments (Table 4 and Figure 2). The *Bifidobacterium* genus and the *Atopobium* cluster significantly decreased, whereas the *Bacteroides* group increased in the feces of the volunteers who received the placebo. These changes did not occur in any of the groups fed probiotic strains (Table 4).

**Table 4:** Bacterial populations in healthy volunteers’ fecal samples by FISH-CF analysis.

Targeted group	Capsule		Time (t)	
		t_1_	t_2_	t_3_
	Placebo	9.6 ± 2.3^ab^	8.7 ± 1.7^a^	5.8 ± 0.8^b^
	*L. rhamnosus*	12.2 ± 2.4	8.3 ± 1.3	9.0 ± 1.3
*Bif164*	*B. breve*	6.3 ± 1.3	6.0 ± 1.3	6.8 ± 1.5
	*B. breve* plus *L. rhamnosus*	8.5 ± 1.8	12.0 ± 3.1	7.2 ± 1.7
	*L. paracasei*	7.3 ± 1.4	7.3 ± 1.3	8.3 ± 1.5
	Placebo	0.2 ± 0.07	0.7 ± 0.3	0.6 ± 0.2
	*L. rhamnosus*	1.0 ± 0.3	0.2 ± 0.1	0.3 ± 0.1
*Erec482*	*B. breve*	0.2 ± 0.1^a^	0.5 ± 0.2^b^	0.2 ± 0.01^ac^
	*B. breve* plus *L. rhamnosus*	0.4 ± 0.1	1.0 ± 0.4	0.3 ± 0.2
	*L. paracasei*	0.3 ± 0.2^ab^	0.3 ± 0.2^a^	1.1 ± 0.3^b^
	Placebo	37.7 ± 3.6^ab^	39.0 ± 2.4^a^	44.6 ± 2.7^b^
	*L. rhamnosus*	42.2 ± 3.1^a^	49.3 ± 3.1^b^	43.0 ± 2.9^ab^
*Clep886*	*B. breve*	44.9 ± 2.6	45.7 ± 3.5	45.5 ± 2.1
	*B. breve* plus *L. rhamnosus*	52.5 ± 3.4	45.1 ± 2.4	47.0 ± 3.7
	*L. paracasei*	46.3 ± 2.4	48.7 ± 3.7	48.7 ± 2.7
	Placebo	12.2 ± 2.1^a^	7.5 ± 1.4^b^	6.7 ± 1.2^bc^
	*L. rhamnosus*	7.9 ± 1.4	7.0 ± 0.9	5.0 ± 0.7
*Ato291*	*B. breve*	6.3 ± 1.3	5.4 ± 0.9	5.9 ± 1.0
	*B. breve* plus *L. rhamnosus*	7.9 ± 2.1	6.1 ± 1.7	6.8 ± 1.5
	*L. paracasei*	4.2 ± 0.8	5.2 ± 0.8	6.2 ± 0.9
	Placebo	16.6 ± 3.2^a^	20.7 ± 2.7^ab^	20.1 ± 2.3^b^
	*L. rhamnosus*	14.6 ± 2.0	15.5 ± 2.1	15.7 ± 2.0
*Bac303*	*B. breve*	20.4 ± 2.6	20.6 ± 2.6	21.7 ± 2.4
	*B. breve* plus *L. rhamnosus*	15.8 ± 2.2	13.7 ± 2.1	18.9 ± 2.8
	*L. paracasei*	17.6 ± 2.0	19.4 ± 2.7	14.1 ± 1.9
	Placebo	0.9 ± 0.2	0.9 ± 0.4	1.4 ± 0.4
	*L. rhamnosus*	1.8 ± 0.5	1.1 ± 0.3	1.3 ± 0.3
*Enter1432*	*B. breve*	1.3 ± 0.4	1.0 ± 0.3	1.3 ± 0.3
	*B. breve* plus *L. rhamnosus*	1.2 ± 0.4	1.1 ± 0.4	1.2 ± 0.5
	*L. paracasei*	0.6 ± 0.2	1.3 ± 0.4	1.7 ± 0.5
	Placebo	3.0 ± 0.5	1.8 ± 0.5	2.5 ± 0.4
	*L. rhamnosus*	2.6 ± 0.5	3.1 ± 0.5	3.6 ± 0.7
*Lab158*	*B. breve*	2.9 ± 0.6	2.5 ± 0.5	2.8 ± 0.6
	*B. breve* plus *L. rhamnosus*	1.8 ± 0.6^a^	3.2 ± 0.8^b^	2.6 ± 0.5^ab^
	*L. paracasei*	2.2 ± 0.4	3.2 ± 0.6	2.9 ± 0.5
	Placebo	5.5 ± 0.8	4.8 ± 0.7	5.5 ± 0.7
	*L. rhamnosus*	5.3 ± 0.8	4.7 ± 0.8	5.7 ± 0.7
*Strc493*	*B. breve*	5.3 ± 0.7	5.1 ± 0.6	6.1 ± 0.8
	*B. breve* plus *L. rhamnosus*	3.7 ± 0.8^a^	5.2 ± 0.7^b^	5.0 ± 0.7^ab^
	*L. paracasei*	3.8 ± 0.6	5.4 ± 0.8	6.2 ± 0.9
	Placebo	6.0 ± 1.0	5.4 ± 0.8	6.4 ± 0.8
	*L. rhamnosus*	6.8 ± 0.6	7.3 ± 1.2	7.8 ± 1.0
*Cdif198 plus Cperf191*	*B. breve*	7.3 ± 1.1	6.8 ± 0.9	7.3 ± 0.7
	*B. breve* plus *L. rhamnosus*	5.5 ± 0.9^a^	7.3 ± 1.1^ab^	9.2 ± 0.6^b^
	*L. paracasei*	6.2 ± 0.9	6.9 ± 1.0	7.2 ± 0.9

Values are means ± SEM, in percentages of living bacteria. n=20 per group. Labeled means without a common letter differ. *P*<0.05. t_1_, first washout; t_2_, intervention; t_3_, second washout.

A transient increase in the *Clostridium coccoides* population occurred at the end of the intervention (t_2_) in those patients fed *B. breve*. However, the percentage of live *C. coccoides* after the second washout returned to initial values (t_1_). The *C. coccoides* population significantly increased with *L. paracasei* administration after the second washout (Table 4).

The *Clostridium leptum* population increased in both the placebo and *L. rhamnosus*-treated groups. However, the increase was continuous in the placebo group, whereas the initial increase in *C. leptum* slightly but significantly dropped with *L. rhamnosus* treatment (Table 4).

Increases in *Lactobacillus* and *Streptococcus* groups were observed in patients fed a mixture of *B. breve* and *L. rhamnosus*. Percentages of live *C. difficile* and *C. perfringens* also increased with administration of the probiotic mixture, but in this case, both *Clostridium* spp. remained elevated after the second washout (t_2_) (Table 4).

Real-time PCR analysis confirmed the increase in *Bacteroides* and the decrease in *Bifidobacterium* spp. observed by FISH-FC in the placebo group (Figure 2C and Figure 2E). *Bacteroides* also increased upon treatment with the mixture of *B. breve* and *L. rhamnosus* (Figure 2C). Interestingly, *C. difficile* increased in volunteers fed *L. rhamnosus* immediately after treatment with this strain ceased (t_2_) (Figure 2B).

*Lactobacillus* significantly decreased in the placebo group after 30 days of intervention and remained low after the second washout. *L. rhamnosus* feeding also resulted in a decrease in *Lactobacillus* at t_2_ (Figure 2D).

As for *Bifidobacterium* spp., this population significantly decreased in those volunteers who received a daily capsule of *L. rhamnosus* when treatment with the probiotic ceased (Figure 2E).

Altogether, these results indicate that both probiotic and placebo administration modified bacterial populations in the volunteers’ feces.

### Fecal strain persistence

A total of 75 colonies from patients fed *L. rhamnosus* CNCM I-4036 were picked from dishes containing MRS modified medium (glucose substituted with rhamnose) and were subsequently grown under anaerobic conditions. DNA analysis by real-time PCR with specific primers for *L. rhamnosus* CNCM I-4036 revealed that 86% of the colonies were positive for this species. This result suggests that at least *L. rhamnosus*, for which there is available specific and selective culture medium, colonized the intestine of volunteers fed this strain for 30 days.

### Fecal secretory IgA content

Secretory IgA content was measured in the stools of the various groups of healthy volunteers (Figure 2A). *B. breve* administration resulted in a significant increase in the fecal secretory IgA content after the 30-day intervention (t_2_), but this increase returned to initial values after the second washout (t_3_). The secretory IgA content did not change in the feces of volunteers who received, *L. rhamnosus*, *L. paracasei* or the *L. rhamnosus/B. breve* mixture.

### White blood cell (WBC) subsets

The effects of probiotic administration on various WBC subsets were analyzed by flow cytometry (Table 5). The most relevant findings were as follows: i) the significant increases in the percentage of CD4+ T lymphocytes and CD4+/CD8+ ratio in the blood of volunteers who received *L. paracasei* and ii) the increase in the percentage of regulatory T lymphocytes observed in the placebo, *L. rhamnosus* and *B. breve* groups. 

**Table 5:** Analysis of immune system populations in volunteers’ blood.

Subset population	Capsule	Time (t)	
		t_1_	t_2_
	Placebo	69.2 ± 1.9	71.4 ± 1.4
	*L. rhamnosus*	70.8 ± 2.5	72.2 ± 1.4
*CD3+(T cells)*	*B. breve*	69.6 ± 3.0	70.1 ± 1.8
	*B. breve* plus *L. rhamnosus*	74.0 ± 2.5	73.3 ± 2.2
	*L. paracasei*	70.5 ± 1.9	72.2 ± 1.2
	Placebo	9.6 ± 0.7	9.8 ± 0.8
	*L. rhamnosus*	10.1 ± 0.5	10.1 ± 0.5
*CD19+(B cells)*	*B. breve*	9.8 ± 0.7	9.5 ± 0.9
	*B. breve* plus *L. rhamnosus*	8.6 ± 0.8	9.6 ± 0.6
	*L. paracasei*	8.7 ± 0.5	8.8 ± 0.7
	Placebo	45.5 ± 1.9	47.6 ± 1.2
	*L. rhamnosus*	46.0 ± 2.1	45.7 ± 1.6
*CD3+ CD4+ (T helper cells)*	*B. breve*	45.4 ± 3.0	47.0 ± 2.3
	*B. breve* plus *L. rhamnosus*	49.5 ± 2.0	50.3 ± 2.0
	*L. paracasei*	43.6 ± 1.7	47.8 ± 1.5^*^
	Placebo	21.3 ± 1.6	22.5 ± 1.1
	*L. rhamnosus*	15.8 ± 2.0	20.3 ± 0.7
*CD3+ CD8+ (T cytolytic cells)*	*B. breve*	20.1 ± 1.6	20.4 ± 0.9
	*B. breve* plus *L. rhamnosus*	20.4 ± 1.8	19.8 ± 1.4
	*L. paracasei*	23.4 ± 1.3	22.4 ± 1.1
	Placebo	2.0 ± 0.2	2.2 ± 0.1
	*L. rhamnosus*	2.1 ± 0.2	2.3 ± 0.1
*CD4+/CD8+ cells*	*B. breve*	2.1 ± 0.2	2.4 ± 0.2
	*B. breve* plus *L. rhamnosus*	2.6 ± 0.2	2.7 ± 0.2
	*L. paracasei*	1.8 ± 0.1	2.1 ± 0.1^*^
	Placebo	4.1 ± 0.2	4.9 ± 0.2^*^
	*L. rhamnosus*	4.0 ± 0.3	4.9 ± 0.3^*^
*CD3+ CD4+ CD25+ CD127- (T regulatory cells)*	*B. breve*	3.8 ± 0.3	5.0 ± 0.6^*^
	*B. breve* plus *L. rhamnosus*	4.5 ± 0.4	5.7 ± 0.8
	*L. paracasei*	4.6 ± 0.2	5.1 ± 0.6
	Placebo	3.1 ± 0.4	4.1 ± 0.5
	*L. rhamnosus*	3.5 ± 0.4	3.6 ± 0.5
*CD14+*	*B. breve*	4.6 ± 0.6	5.3 ± 0.7
	*B. breve* plus *L. rhamnosus*	3.6 ± 0.6	3.9 ± 0.4
	*L. paracasei*	2.8 ± 0.5	3.9 ± 0.6

Results are mean ± SEM, as percentage of total accounted cells. n=20 per group. *P<0.05. t_1_, first washout; t_2_, intervention.

### Cytokine concentrations in volunteers’ serum

**Figure 3:**
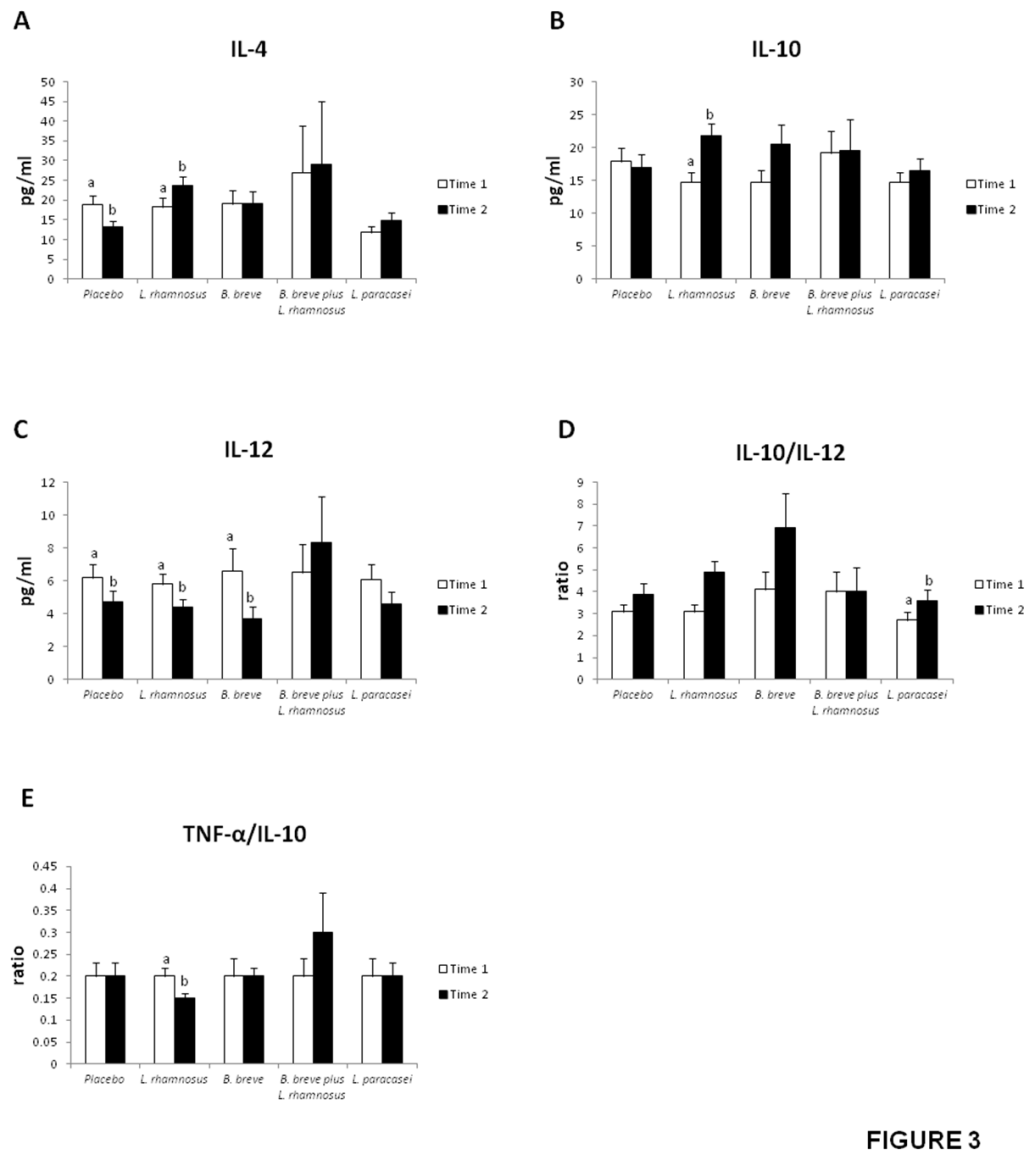
Serum IL-4 (A), IL-10 (B), and IL-12 (C) concentrations and IL-10/TNF-α (D), and IL-10/IL-12 ratios (E) in healthy adults fed one daily capsule of probiotics or placebo for 4 weeks. Values are means ± SEM, n=20 per group. Labeled means without a common letter differ, *P*<0.05. Time 1, first washout; Time 2, intervention.

Serum IL-4, IL-10 and IL-12 concentrations, as well as the IL-10/IL-12 and TNF-α/IL-10 ratios appear in Figure 3. All patient groups showed similar values of IL-6, TNF-α and TGF-β (not shown).

Whereas the anti-inflammatory cytokine IL-4 decreased in the group fed the placebo for 30 days, the serum concentration of this cytokine remained unchanged in the groups that received *B. breve*, *L. paracasei* or the combination of both. In contrast, IL-4 increased in those volunteers fed *L. rhamnosus* (Figure 3A). The latter probiotic strain also increased the concentration of another anti-inflammatory cytokine, IL-10 (Figure 3B).

Volunteers fed the placebo, *L. rhamnosus* or *B. breve* exhibited significantly lower values of the pro-inflammatory cytokine IL-12 at the end of the intervention (t_2_) compared with baseline (t_1_) (Figure 3C). The IL-10/IL-12 ratio, an anti-inflammatory index, significantly increased in patients who received *L. rhamnosus* and *L. paracasei* (Figure 3D). In contrast, *L. rhamnosus* treatment decreased the TNF-α/IL-10 ratio, a pro-inflammatory index (Figure 3E).

Altogether, these findings point to a clear immunomodulatory effect of the three probiotic strains, with *L. rhamnosus* exerting the most robust effect.

## Discussion

In this study, the safety, tolerance, persistence and effects on the immune system of the probiotic strains *Lactobacillus paracasei* CNCM I-4034, *Bifidobacterium breve* CNCM I-4035 and *Lactobacillus rhamnosus* CNCM I-4036 [10] were investigated in 100 healthy volunteers. We found that the recorded gastrointestinal symptoms (GSRS and daily recorded symptoms), defecation frequency and stool consistency were not altered by probiotic intake in healthy volunteers. Moreover, no relevant changes in blood and serum parameters, and no adverse events occurred during and after treatment. All three probiotic strains were sensitive to ampicillin and tetracycline.

Probiotic administration modified bacterial populations in the volunteers’ feces as evidenced by real-time PCR and fluorescence *in situ* hybridization. Some of the alterations were transient, whereas others were stable. The most relevant finding regarding bacterial populations was the increase in *Clostridium difficile* that took place in feces when *L. rhamnosus* CNCM I-4036 administration ceased (t_3_, Figure 2B), which points to a clear beneficial effect by this probiotic strain. Volunteers may have experienced a displacement of *C. difficile* by *L. rhamnosus* CNCM I-4036 during the intervention of 30 days. Many studies have shown a decrease in *C. difficile* adhesion to intestinal mucosa by probiotics [27-29].

The fact that total bifidobacteria counts were reduced in the group treated with *B. breve* CNCM I-4035 suggests that the administered strain either did not reach the colon in a viable state in significant numbers or did not proliferate in the colon. Bifidobacteria counts also decreased by *L. rhamnosus* CNCM I-4036 feeding when t_3_ is compared with t_1_ (Figure 2E).

Strikingly, certain bacterial populations changed in the feces of volunteers in the placebo group. This effect caused by the placebo might be due to the cow’s milk and/or sucrose included in its composition. The fact that regulatory T lymphocytes were increased in the placebo group is also intriguing. The same is true for the observation that IL-4 was decreased in the placebo group. This may indicate that the sample size, while calculated prior to start of the trial for the main outcome, was too low for these two variables.

Interestingly, *L. rhamnosus* CNCM I-4036 was identified after the intervention (t_2_) in fecal samples of volunteers that received this bacterial strain. This finding does not necessarily imply successful colonization but rather persistence of the strain at this time period. Detection of *L. rhamnosus* CNCM I-4036 for a much longer period would be needed to determine whether the strain does in fact colonize the gastrointestinal tract. Also, high-throughput sequencing techniques would be helpful. Persistence in feces by the two other assayed strains, *L. paracasei* CNCM I-4034 and *B. breve* CNCM I-4035, could not be proven due to the lack of specific and selective culture media.

Another interesting finding of this work was that the *B. breve* CNCM I-4035 administration resulted in a significant increase in secretory IgA content after the 30-day intervention. After the second washout, this increase returned to initial values, which points to a clear effect due to the probiotic. This result confirms previous results from our group [10]. We have reported that *B. breve* CNCM I-4035 led to a higher IgA concentration in both feces and plasma of mice [10]. Modification of secretory IgA has a clear and important effect on the immune system. Secretory IgA serves as the first line of defense in protecting the intestinal epithelium from enteric toxins and pathogenic microorganisms (30). Secretory IgA promotes the clearance of antigens and pathogenic microorganisms from the intestinal lumen by blocking their access to epithelial receptors, entrapping them in mucus, and facilitating their removal by peristaltic and mucociliary activities [30].

It has been suggested that the safety of probiotics should be further evaluated by the detection of undesirable changes in immune parameters [31] because of growing evidence that probiotics, especially lactobacilli and bifidobacteria, have immunomodulatory properties. The main finding of our cytokine analysis was that *L. paracasei* CNCM I-4034, *B. breve* CNCM I-4035 and *L. rhamnosus* CNCM I-4036 exerted immunomodulatory effects. Increased levels of anti-inflammatory molecules (IL-4, IL-10, IL-10/IL-12) and decreased levels of the pro-inflammatory index (TNF-α/IL-10) were found in the serum of volunteers fed *L. rhamnosus* CNCM I-4036. IL-12 also decreased in volunteers that received *B. breve* CNCM I-4035, whereas the anti-inflammatory index (IL-10/IL-12) increased in the group fed *L. paracasei* CNCM I-4034. Immunomodulation by these three probiotic strains has been reported in *in vitro* experiments by Bermudez-Brito et al. [32,33]. These authors showed that *L. paracasei* CNCM I-4034, *B. breve* CNCM I-4035 and *L. rhamnosus* CNCM I-4036 inhibited the production of pro-inflammatory cytokines and chemokines by human intestinal dendritic cells challenged with pathogenic bacteria and that such an effect seems to be mediated through a decreased expression of toll-like receptor (TLR)-1, TLR-5 and TLR-9 [32,33].

In summary, our results demonstrate that the intake of the three bacterial strains was safe and exerted a varying degree of immunomodulatory effects. In particular, *L. rhamnosus* CNCM I-4036 colonized the intestine, and *B. breve* CNCM I-4035 enhanced production of intestinal secretory IgA. Our findings also confirm previous results obtained in mice. Overall, these results warrant further studies and open the possibility of undertaking similar trials in patients affected by intestinal pathologies.

## Supporting Information

Checklist S1 **CONSORT Checklist.**
(DOC)

Protocol S1 **Trial Protocol.**
(DOC)

Table S1 **Probes used in fluorescence in situ hybridization (FISH).**
(DOCX)

Table S2 **Primer sequences used in real-time PCR.**
(DOCX)

Table S3 **Frequency and consistency of the feces.**
(DOCX)

Table S4 **Percentage of antimicrobial resistance populations.**
(DOCX)
