# Supplementary material for: Safety and Immunomodulatory Effects of Three Probiotic Strains Isolated from the Feces of Breast-Fed Infants in Healthy Adults: SETOPROB Study
Source: PLoS One. 2013 Oct 28;8(10):e78111. doi: 10.1371/journal.pone.0078111 (PMC3810271; doi:10.1371/journal.pone.0078111)
Supplement: Protocol S1 — Trial Protocol. (DOC) [file pone.0078111.s002.doc]

No protocol / no review CT09-November 001/39

**TITLE: Colonization, SECURITY, AND EFFECTS ON TOLERANCE IMMUNE SYSTEM OF THREE ADULT HEALTHY probiotic strains.**

**PROBIOTICS WELFARE AND HEALTH HERO**

**ACRONYM**: SETOPROB

**KEYWORDS**: Probiotics, adults, tolerance, security, lactobacilli, bifidobacteria.

**DATE**: 14/04/2011

Last Revision: 13/06/2011

INVESTIGATOR:

Dr. Peter Abellán Crossbow

pedro.abellan @ hero.es

Director of Quality and Development

Hero Spain, SA

Hero Institute for Infant Nutrition

Tel 968898900

Fax. 968898952

No protocol / no review CT09-November002/39

1. CONTENTS

1. INDEX ................................................. .................................... 2

2. PARTICIPANTS ................................................. ....................... 3

3. PROJECT SUMMARY .............................................................. 5

4. INTRODUCTION ................................................. ....................... 7

5. WORKING HYPOTHESIS

5.1. Null hypothesis ................................................ ....................... 13

5.2. Alternative Hypothesis ..................................................................... 13

6. OBJECTIVES.

6.1. General ................................................. ........................... 13

6.2. Specific ................................................. ........................ 13

7. METHODOLOGY

7.1. Subjects ....................................................................................... 14

7.2. Experimental design ................................................................ 16

7.3. Main outcome ......................................... 17

7.4. Study period ............................................... ............. 18

7.5. Treatment Overview ...................................................... 19

7.6. Study development .............................................................. 19

7.7. Ethical ...................................................................... 21

7.8. Sampling and Analytical Methodology .............................. 21

8. ANALYTICAL DATA COLLECTION .......................................... 33

9. ACTIVITY OR TASK ................................................................ 34

10. MILESTONES………………………………………………………………………… 37

11. SCHEME AND WORK PLAN ..................................................... 37

12. CONTRIBUTION TO STRATEGIC PURPOSE .................................. 38

13. OR RISKS ANTICIPATED CONTINGENCIES ............................... 38

14. ECONOMIC REPORT ................................................................ 38

15. APPENDICES .............................................................................................. 39

No protocol / no review CT09-November 03/39

2. PARTICIPANTS

Research groups involved.

Dr. Peter Abellán Crossbow *

pedro.abellan @ hero.es

Dr. Fernando Romero Braquehais

fernando.romero @ hero.es

Dr. Maria José Bernal Cava

mjose.bernal @ hero.es

Rosario Martinez Chair

rosario.martinez @ hero.es

Immaculate Ortuño Diaz

inmaculada.diaz @ hero.es

Dr. Francisco Saez Soto

paco.saez @ hero.es

Luis Manuel Sanchez-Siles

luisma.sanchez @ hero.es

Esther Matencio Hilla

esther.matencio @ hero.es

Hero Institute for Infant Nutrition

HERO SPAIN, SA

Avenida de Murcia 1

30,820 Murcia

Tel: +34 968898900

Fax: +34 968 898952

Dr. Ángel Gil Hernández *

agil@ugr.es

Dr. Carolina Gómez Llorente

Julio Plaza

Miriam Bermudez Brito

Department of Biochemistry and Biology

Molecular II

Institute of Nutrition and Technology

Food

Biomedical Research Center

University of Granada

Health Campus

Armilla 18100, Granada, Spain

Tel +34 958241000 ext 20307

Fax +34 958819132

Mobile +34695466922

Dr. Gaspar Ros Berruezo *

gros@um.es

Dr. M ª Jesús Periago Castón

Dr. Carmen Martinez Graciá

Dr Francisco Javier Garcia Alonso

Dr. Carmen Frontela Saseta

Patricia Weight Echarri

Scholar

008-02 Research Group

Technology Department, Nutrition and

Food Science

Faculty of Veterinary

University of Murcia (UMU)

Espinardo

30,071 Murcia

H.Phone 868884794

Mobile 649444603

Internal extension 2312

* IP (Principal Investigator)

Dr. Daniel Ramón Vidal *

daniel.ramon @ biopolis.es

Genoese Salvador Martinez

salvador.genoves @ biopolis.es

Dr Empar Chernoll

empar.chernoll @ biopolis.es

BIOPOLIS, SL

C / Professor Augustine Escardino 9

building B2

University of Valencia Science Park

46980-Paterna, Valencia

SPAIN +34 963 160 299

Dr. Dolores Corella Piquer *

Preventive Medicine and Public Health,

Food Science,

Toxicology and Forensic Medicine.

Avenida Blasco Ibáñez, 15

Faculty of Medicine, Level 1-E

46010-Valencia

H.Phone (Secretariat) 963 864 417

No protocol / no review

CT09-November 01 4/39

COORDINATOR

Esther Matencio Hilla

Hero Institute for Infant Nutrition

Research Department

Hero Spain, SA

Avenida de Murcia, 1

30820, Alcantarilla

Murcia

Tel: +34 968898900 Ext 1166

Fax: +34 968 898952

esther.matencio @ hero.es

No protocol / no review CT09-November 05/39

3. PROJECT SUMMARY.

**Introduction**

The intestinal microbiota and its importance in the health and welfare of individuals think one of the research lines developed in recent decades. Has shown that individuals

Allergy obese and have an intestinal microbiota differently from healthy individuals. The Hero Child Nutrition Institute, has developed together with the Department of Biochemistry the University of Granada, a draft isolation and characterization of probiotic strains to from feces of infants exclusively breastfed. These strains have been registered at the Institut Pasteur (France) and patented. Controls have surpassed toxicology and safety standards in the report of FAO / WHO, and its functionality as inhibition Pathogen growth and modulating the immune system has been tested in assays in vitro and in vivo in murine and cellular models.

This report details the design of a clinical trial in healthy adults to test whether the Daily consumption of the new strains Hero property contributes to intestinal colonization is safe and tolerable, and can contribute to health and well-being of healthy individuals.

**Hypothesis**

Daily administration of one or more of the probiotic strains, (CNCM I-4034, CNCM I-4035, CNCM I-4036), colonizes the intestinal microbiota in healthy adults is safe, well tolerated and Regular consumption has positive effects on gastrointestinal and immune system.

**Methods**

There will be a randomized, double-blind, randomized, placebo-controlled, multicenter 4 groups of study and control group.

• Colonization of strains and modification of the intestinal microbiota assessed through identifying strains by RT-PCR with specific probes and populations counts selected bacterial immunofluorescence in situ (FISH).

• The safety of consumption of the strains was assessed by physical examination, the parameters Blood and stool tests of ampicillin and tetracycline resistance in lactic flora.

• Tolerance was assessed by recording of gastrointestinal symptoms and registration

appearance and stool frequency.

• The effect on systemic and adaptive immune system is measured through measures

blood lymphocyte populations and cytokines in plasma, serum IgA, saliva and feces. AGCC

in feces.

**Expected Results**

• They expect to see changes in the intestinal microbiota microbiota towards a more percentage of lactobacilli and bifidobacteria and is expected a decrease in enterobacteria

No protocol / no review CT09-November 06/39

and clostridia. It is also expected that there are no changes in susceptibility of strains original to change their susceptibility to antibiotics.

• It is expected that the hematological parameters as well as the appearance and frequency of the stool change relative to controls and all of them coming within normal ranges

• It is expected that these probiotics influence the lymphocyte response, being able to foresee an possible strains immunomodulatory effect in diseases such as allergies, IBD syndrome metabolic.

• This effect would have to be tested in future studies in subjects with any of the diseases mentioned.

No protocol / no review CT09-November 07/39

4. INTRODUCTION

The supplementation of foods with functional ingredients is a widely used technique food industries by those whose goals are not limited to food production insurance and nutritional, but go beyond, wanting to contribute to the welfare and health of the consumers. During the last two decades, the number of functional foods on the market has shot and not always healthy nutrition claims found in the labeling has been properly tested. That is why in 2006 enters into force European Regulation on Nutrition and Health Claims in order to control the false allegations massive increase in food and consumer protection.

Probiotics, live microorganisms administered in adequate amounts which produce a benefit to the host (WHO / FAO 2001-2002), are subject to a safety assessment and functionality before being introduced into the human food. Once tested using in vitro and in vivo safety and viability of the strains, the group of work of FAO / WHO requires the completion of a human clinical trial phase I in order to check the security of different probiotic strains before release on the market. In this document, it is proposed to conduct the second human clinical trial, double blind, randomized trial to test the safety, tolerability and efficacy of the strains under study, security being established from hematological data and physical examination and tolerance from the adverse effects questionnaires and stool frequency and (Sierra et al., 2010; Wind et al., 2010). After the ingestion of probiotic strains if the data are observed hematological remain in the normal range throughout the study and no Differences between groups (control and probiotic) or the differences between the time in the same group, the physical examination is optimal and volunteers do not experience adverse effects, confirm the safety and tolerance of the strains consumption (Sierra et al., 2010; Wind et al. 2008).

Numerous health disorders and gastrointestinal tract infections, allergies, obesity, antibiotic-induced diarrhea, have been associated with altered intestinal microbiota (Kalliomaki et al., 2008; (Chmielewska and Szajewska, 2010). High levels of bifidobacteria in the intestinal microbiota have been associated with reduced gastrointestinal infections (Hooper, 2004). Bacterial groups count in feces, indicating whether a probiotic treatment can modify the intestinal microbiota to a healthy intestinal flora. Increased Bifidobacteria and / or fecal lactabacillos along with an increase in the content of AGGC and defecation frequency could indicate the utility of probiotics in the treatment of constipation, diarrhea, etc ...

Probiotic bacteria produce short chain fatty acids, AGCC, lowering pH intestinal, favoring the growth and survival of groups such as bifidobacteria and eradicating some pathogenic bacteria. The AGGC are energy source for colonocytes, influencing the lipid and carbohydrate metabolism, control the pH of the colon, maintain intestinal mucosal integrity, motility and absorption. Have shown that diet fermented products restriction leads to a decrease in fecal SCFA (Olivares et al., 2006) and SCFA production is dependent on the properties fermentation for each strain.

There are numerous studies that have demonstrated the immunomodulatory effect of different Probiotics (Klein et al. 2008; Ouwehand et al., 2008). Some of these studies show correlation between changes in immune parameters and protective effects against Common infectious diseases of the respiratory and / or gastrointestinal (Vrese et al 2005; Guillemard et al., 2010)

IgA is the main immunoglobulin responsible for defense against pathogens. Increasing IgA in feces has been linked to anti-infective properties of probiotics in diarrheal diseases. And increased presence of salivary IgA indicates that the probiotic has a potentiating effect of

No protocol / no review CT09-November 08/39

defense against microorganisms, which is not limited to the gastrointestinal gastrointestinal but extends to other mucosal (Lara-Villoslada et al., 2007). Increased IgA, IgM and total IgG in plasma, may cause the activation of a specific immune response which can improve the response to infections (Sierra et al., 2010).

C-reactive protein, CRP, is also a marker of inflammation easy to measure the potential anti-inflammatory probiotics and other substances.

Increasing regulatory cytokines has been linked to the immunomodulatory effects of the probiotic strains in pathologies such as allergies or inflammatory bowel disease (Felezsko et al. 2007; Smits et al., 2005). TNF-α is an inflammatory mediator that acts by activating various cells.

Interleukin IL-2 is important in the inflammatory response and viral infection, activated NK cells and induces activation and proliferation of lymphocytes T. Can be an important factor in the struggle of probiotics against respiratory infection.

Sierra et al. studied the effect on the immune system and gastrointestinal daily intake of Lactobacillus salivarius for 4 weeks in a group of healthy adults. Plasma found levels of TGF-β and IL-10 (regulatory cytokines) and TNF-α (Th1 cytokine) increase. It same with monocytes and natural killer cells, a fact that shows like consumption of certain probiotic strains affect inmnune innate and specific response, the monocytes and macrophages with the dendritic cells play a crucial role in the response Innate against microbial antigens, leading to the fact that activation of the immune system adaptive. The increased TNF-α and increased IL-10, TGF-β activation evidenced immune system. The emergence of these regulatory cytokines could be a potential factor in the homeostasis of the immune system, since they are involved in T lymphocyte response and TH3 regulators. IL-10 and TGF-β have been linked to phenomena of tolerance and control various immune processes including inflammation, allergy or infection (Sierra et al., 2010).

In recent years, besides the role of probiotics in the treatment of diseases as inflammatory bowel disease, Crohn's disease and diarrhea, research this leading to the role of probiotics and prebiotics in the prevention and / or treatment of metabolic syndrome. The intestinal microbiota of healthy microbiota differs obese and Allergy (Collado et., 2008; Kalliomaki et al., 2008). Canine et al. in mice fed high fat diets and therefore obese plasma levels observed in GLP-1 (regulator of food intake and glucose) and GLP-2 (regulator of intestinal endotoxemia) lower mice that were fed the same diet supplemented with oligofructose (Cani et al. 2007).

This research group showed that the intake of prebiotics could improve glucose tolerance, insulin secretion and reduce the levels of dietary intake, observed a correlation between the increase of bifidobacteria and decreasing weight and endotoxemia in the intestine.

Martin et al. demonstrating that probiotics to cause a change in the intestinal microbiota could be modulating lipid metabolism and bile salts and altering fat distribution bowel, in germ-free mice colonized with human infant microbiota *L.paracasei* supplemented or *L.rhamnosus* (Martin et al., 2008)

Appears to modulate the gut microbiota energy balance through a number of mechanisms. A change of the intestinal microbiota (probiotics addiction) might be a good strategy to modify the energy balance.

No protocol / no review CT09-November 09/39

Since the entry into force of the European regulation on nutrition and health claims, numerous companies have submitted dossiers to EFSA with valuable evidence for support arguments relating to the use of probiotics in the prevention of diseases (Valio, DANONE, NUMICO). The agency has not yet approved any claim related to the use of probiotics. In December 2010, EFSA held a meeting to clarify the criteria that were used in the evaluation of the claims related to the immune system and the gastrointestinal tract. The information provided by the agency, was scarce. The only conclusions could be extracted and must be taken into account in the design of those trials Clinical intended to be addressed to new probiotic strains, were as follows:

Arguments could be well defined:

- contribute to the reduction of the presence of pathogens or their toxins in the intestine.
- contribute in defense against intestinal pathogens.
- They help reduce risk factors for gastrointestinal infections.
- The following arguments are considered beneficial physiological effects by the panel:
- Increased number of bifidobacteria or lactobacilli.
- Decreased number of diners (clostridia, enterobacteria, Bacteroides, etc.)
- An increase or decrease in the number of specific microorganisms in the gastrointestinal tract,
- it is a beneficial physiological effect alone.
- Benefits are the following claimed effects:
- Reduced number of pathogens or their toxins in the gastrointestinal tract.
- Reducing risk factors of gastrointestinal diseases.
- The presence of pathogenic microorganisms or toxigenic in the gastrointestinal tract can
- lead to infection. Panel believes that reducing the number of these pathogens
- specific or their toxins, may be a beneficial physiological effect.
- The variables to measure the reduction of gastrointestinal pathogens and infections are:
- Reduced number, proportion or prevalence of pathogens
- Reduction toxins.
- Clinical variables (duration, incidence, severity of symptoms) and other variables (pH, SCFA, intestinal permeability ...)

No protocol / no review CT09-November 10/39

Pathogens could be gastrointestinal pathogens (H. pylori, Clostridium difficile ...) and / or pathogens or toxins in the food chain (Salmonella, S.aureus, E.sakazakii) The pathogen testing methods recommended are the classical methods, molecular (FISH, RT-PCR, DNA arrays, DNA sequencing), immunologic (ELISA, etc ...)

Groups accepted study population: General population, high susceptibility groups infections (athletes, stress), populations with high incidences of infections. It is not possible extrapolate from children to adults.

The Hero Child Nutrition Institute, in its scientific program has isolated three different strains from faeces of healthy infants fed through exclusive breastfeeding. These strains have been registered at the Pasteur Institute and patented. Sequencing strains genomic and toxicological tests (hydrolysis of bile salts, isomers DL lactic acid, polyamines, antibiotics resistance tests and animal tests intake inmnune both immune-competent and-depressed) confirmed the safety of the strains (See Appendix 0). According to clinical trials for safety and tolerability of probiotic strains consulted and recent comments by the EFSA, the Institute has identified various variables relating to the safety, tolerability and functionality for carrying strains of this clinical trial.

Bibliography

• Joint FAO / WHO Working Group Report on Drafting Guidelines for the Evaluation of Probiotics in Food London, Ontario, Canada, April 30 and May 1, 2002.

• Scientific Opinion of the Panel on Biological Hazards on a request from EFSA on the maintenance of the list of QPS microorganisms intentionally added to food or feed. The EFSA Journal (2008) 923, 1-48.

• Kekkonen RA, Lummela N, Karjalainen H, Latvala S, Tynkkynen S et al (2008). Probiotic intervention has strain-specific anti-inflammatory effects in healthy adults. World J Gastroenterol 2008 April 7, 14 (13): 2029-2036

• Honeycutt, TCB, Khashab He, M., Wardrop, RM, McNeal-Trice, K., Honeycutt, ALB, Christy, C. G., Mistry, K., Harris, B. D. and Meliones, J.N., 2007. Probiotic administration and the No incidence of nosocomial infection in pediatric intensive care: A randomized placebo controlled trial. Pediatr. Crit. Care Med.8, 452-458.

• Zein, EF, Karaa, S., Chemaly, A., Saidi, I., Daou-Chahine, W., Rohban, R., 2008. [Lactobacillus rhamnosus septicemia in a diabetic patient Associated with probiotic use: a case report]. Annales de Biologie Clinique 66, 195-198.

• Hooper LV (2004). Contributions to mammalian gut bacterial development. Trends Microbiol 12: 129-134

• From Vrese M, Winkler P, Rautenberg P, Harder T, Noah C et al (2005). Effect of Lactobacillus gasseri PA 16/8, Bifidobacterium longum SP 07/3, B. bifidum MF 20/5 on common cold episodes: A double blind, randomized, controlled trial. Clinical Nutrition 24, 481-491

No protocol / no review CT09-November 11/39

• Guillemard E, Tondu F, F and Lacoin Schrezenmeir J (2010). Consumption of a fermented dairy product container containing the probiotic Lactobacillus casei DN-114 001 you reduce the duration of respiratory infections in the elderly in a randomized controlled trial. British Journal of Nutrition 103, 58-68

• Klein A, Friedrich U, Vogelsang H, et al. (2008) Lactobacillus acidophilus 74-2 and Bifidobacterium animalis subsp. DGCC lactis 420 modulate unspecific cellular immune response in healthy adults. Eur J Clin Nutr 62, 584-593.

• AC Ouwehand, Bergsma N, Parhiala R, et al. (2008) Bifidobacterium microbiota and parameters of immune function in elderly subjects. FEMS Immunol Med Microbiol 53, 18-25.

• Sierra S, Lara-Villoslada F, Sempere L, Olivares M, Boza J, Xaus J (2010). Intestinal and immunological effects of daily oral administration of Lactobacillus salivarius CECT5713 to healthy adults. Anaerobe 16: 195-200

• Feleszko W, Jaworska J, Rha RD, Steinhausen S, Avagyan A, Jaudszus A, et al. (2007). Probiotic-induced suppression of allergic sensitization and airway inflammation is Associated With An Increase of Tregulatory-dependent mechanisms flag in a murine model of asthma. Clin Exp Allergy 37:498 E505.

• HH Smits, Engering A, vanderKleij D, deJong EC, Schipper K, vanCapel TM et al. (2005). Selective probiotic bacteria induce IL-10-producing regulatory T cells in vitro by modulating dendritic cell function through dendritic cell-specific intercellular adhesion molecule3-grabbingnon integrin. J Allergy Clin Immunol 115:1260 e7.

• Olivares M, Diaz-Ropero MP, Gomez N, Sierra S, Lara-Villoslada F, Martin R, et al. (2006). Dietary deprivation of fermented foods causes a fall in innate immune response. Lactic acid bacteria can counteract the immunological effects of this deprivation. J Dairy Res 73:492 e8.

• Chmielewska A and Szajewska H. (2010). Systematic review of randomized controlled trials: Probiotics for functional constipation. World J Gastroenterol 2010 January 7, 16 (1): 69-75

No protocol / no review CT09-November 12/39

5. WORKING HYPOTHESIS.

5.1. Null hypothesis.

Daily administration of one or more of the probiotic strains, (CNCM I-4034, CNCM I-4035, CNCM I-4036) in healthy individuals colonizes the intestinal microbiota, not safe, not well tolerated, and gastrointestinal and immunological effects.

5.2. Alternative hypothesis.

Daily administration of one or more of the probiotic strains, (CNCM I-4034, CNCM I-4035, CNCM I-4036), colonizes the intestinal microbiota is safe, well tolerated and has effects Gastrointestinal and immunological.

6. OBJECTIVES.

6.1. General.

The main objective is to evaluate the daily administration of one or more of the strains probiotic, CNCM I-4034, CNCM I-4035, CNCM I-4036, colonizes the intestinal microbiota healthy adults is safe, well tolerated.

Secondary objectives, gastrointestinal and immunological effects of consumption of the strains (CNCM I-4034, CNCM I-4035 and CNCM I-4036) for healthy individuals.

6.2. Specific.

Evaluate strains colonizing the intestine of healthy subjects by RT-PCR.

Evaluate the safety of the strains through measures leukocyte number, percent Neutrophils, blood pressure, heart rate, detecting ampicillin resistant lactic flora and tetracycline.

No protocol / no review CT09-November 13/39

Assess tolerance isolates by recording symptoms and gastrointestinal discomfort systemic symptoms (fever, headache, muscle and bone pain, symptoms flu) and recording aspect and stool frequency of volunteers during study period.

Count bacterial groups (lactobacilli, bifidobacteria, clostridia, Bacteroides) by RT-PCR, plate count bacteria (bifidobacteria, lactobacilli and L.rhamnosus) and genus Bifidobacterium, Bacteroides group, Enterobacter, Streptococcus group, Lactobacillus group, Atopobium cluster, Clostridium coccoides group, Clostridium leptum group, Clostridium perfringens and Clostridium difficile spp. by FISH.

Assess administration Hero strains have beneficial effect level gastrointestinal and possible effects on the immune system against microorganisms pathogens, through measures IgA, pH, moisture and fecal SCFA.

Check possible effects of the three strains in the immune system through measures IgA saliva and plasma, plasma measures ultrasensitive CRP, cytokines (IL-10, IL-12, IL-4, IL-6, TNF-α, TGF-β,) and blood lymphocyte population (T cells (CD3 +), B lymphocytes (CD19 +) cells, T helper (CD4 +), cytotoxic T lymphocytes (CD8 +), suppressor T cells (CD4 + CD25 +), NK cells (CD56 +) and monocytes (CD14 +)).

7. METHODOLOGY.

7.1. Subjects.

Eligibility for inclusion.

Healthy adults (18-50 years).

Normal defecation.

Normal blood parameters.

BMI (18 - <30).

Exclusion conditions.

Administration of antibiotics and / or gastrointestinal disorders 1 month before the study.

Pregnancy and lactation.

Drug-related gastrointestinal diseases.

No protocol / no review CT09-November 14/39

Diarrhea.

Constipation (less than 3 bowel movements per week).

Diabetes.

Blood pressure> 130/90 mmHg

Metabolic diseases.

Allergies routinely treated.

Allergy to cow's milk.

Lactose intolerance.

Smoking.

Requirements of the volunteers during the study.

• Daily consumption over 4 weeks of a capsule at breakfast.

• no food in the diet with probiotics and / or prebiotics throughout the period of study (not Actimel, Activia, etc ...) (see list in Annex 6)

• Daily booking and general gastrointestinal symptoms, frequency and appearance of depositions in a notebook for data collection.

• Annotation of a weekly 24-hour recall questionnaire.

• Attend 3 visits scheduled for control and monitoring of the study.

• Delivery of faecal samples and saliva (elapsed 2 weeks, 6 weeks and 8 weeks from the

beginning of test).

• Blood tests (after 2 and 6 weeks from the start of the trial)

Criteria for withdrawal.

• Insufficient compliance protocol, defined as: not go to study visits, not provides the data that are recorded in food diaries / tolerance. Intake foods that can influence the results of the test product.

No protocol / no review CT09-November 15/39

• Vomiting, diarrhea and / or constipation to be caused by the new product.

• Occurrence of some organic disease, systemic or otherwise that may prevent evaluate the response of the study.

• Taking antibiotics.

• Voluntary.

It will replace all subjects whose cases have been described as "Defaulting protocol ". Be recorded in Annex 4 (participant data sheet), the reasons that led to the withdrawal from the study.

Calculating the number of subjects.

The sample size is calculated based on the primary endpoint, colonization of the strains in the intestinal microflora (log strain cfu / g). To ensure study power of 90% and risk of 5% need 18 subjects per group. Propose five volunteer groups. In this number must add approximately 10% in anticipation of possible cases of abandonment or withdrawal corresponding to 20 per group and 100 subjects.

7.2 Experimental Design.

Type of clinical trial.

A clinical, randomized, controlled (placebo and control group), for the three strains under study. The clinical study will be developed according to the criteria established in the Declaration of Helsinki and clinical trials process Hero Spain SA


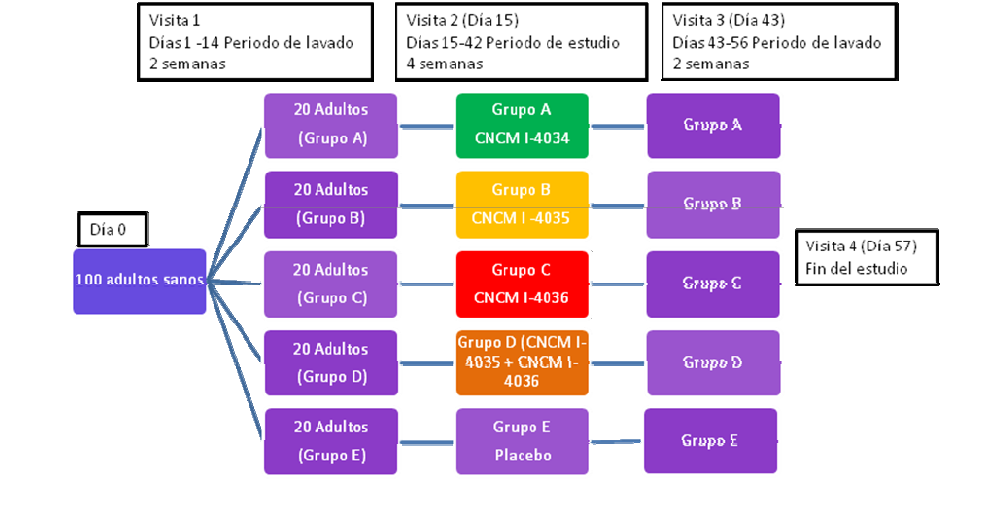


No protocol / no review CT09-November 16/39

7.3. Main study variables.

The primary endpoints of this study are:

• security-related variables: number of leukocytes, percentage of neutrophils, stress blood pressure, heart rate, lactic flora detection ampicillin and tetracycline resistant.

• Variables related to tolerance: systemic symptoms (fever, headache, pain in musculature ...), gastrointestinal symptoms (GSRS scale), stool consistency (King's Stool Chart) and frequency of defecation.

• Variables related to the colonization of the strains in the intestinal microflora.

The main secondary endpoints are:

• IgA in feces, saliva and serum.

• AGCC, moisture and pH and enzymatic activity in feces (APIzym strip system)

No protocol / no review CT09-November 17/39

• Total number of bacteria, microbial composition (FISH, RT-PCR, plate cultures)

• Measurements of plasma cytokine (IL-10, IL-12, IL-4, IL-6, TNF-α, TGF-β, IL12/IL-10, TNF-α/IL-

10).

• Measures percentages blood T lymphocytes (CD3 +), B lymphocytes (CD19 +) T lymphocytes helper (CD4 +), cytotoxic T lymphocytes (CD8 +), suppressor T cells (CD4 + CD25 +) cells NK (CD56 +) and monocytes (CD14 +).

• Clinical examination (weight, height, BMI, blood pressure, heart rate)

• haematological parameters (red blood cells, hemoglobin, hematocrit, MCV, MCH, MCHC, leukocytes, segmented neutrophils, eosinophils, basophils, lymphocytes, platelets), function liver (AST, ALT, GT), kidney (creatinine), others (cholesterol, HDL-cholesterol, cholesterol: HDL-cholesterol ratio, glucose, mean corpuscular volume MCV, WBC), TG, glucose and insulin.

7.4 Period of the study.

Calendar and expected completion date.

The study was initiated after approval by the appropriate ethics committee. For each one of the strains of Hero inclusion period is 8 weeks.

The study will last approximately 16 weeks. The study could be extended by another 4 weeks removal of individuals and need to recruit new patients or problems recruitment, which would end 20 weeks after it began.

**Recruitment assay scheme** Day 1 Day 1 Day 14 Day 43 Day 56

Consent informed X

Compliance with the

requirements of the study X

Delivery of capsules X

Delivery CRDs X X X

Recovery CRDs X X X

**Evaluations**

Clinical test X X X

Alimentation record X X X

Stool samples X X X

No protocol / no review CT09-November 18/39

Salive samples X X X

Blood samples X X

7.5 Description of treatment.

Power of the study.

The product under study is administered in the form of lyophil inside a capsule.

Capsule containing placebo Code: PRO0

Capsule containing 4034 CNCM I-Code: PRO4

Capsule containing CNCM I-4035 Code: PRO5

Capsule containing CNCM I-4036 Code: PRO6

CNCM capsule containing I-4035/36 Code: PRO56

The daily dose of each will be approximately probiotic 1010 cfu / day. The composition of the capsules and the final content of the strains was attached in ANNEX I.

Daily Dosage.

The intake of the capsule will take place at breakfast, preferably with a glass of water.

In the Data Collection Booklet volunteer (ANNEX VI) includes a list of food with probiotics and / or prebiotics that should not be consumed during the 8-week study.

No protocol / no review CT09-November 19/39

7.6 Development of the study.

Recruitment of individuals. Conduct a briefing on clinical trial each center involved (UMU, UGR, UV). During this meeting, will be given sheets ANNEX II volunteer information and summon those interested in participating in the trial for the blood and anthropometric measures in the 15 days following the meeting, period during which must comply with dietary restrictions.

Only when you get the informed consent form shall be the beginning of the ANNEX III study.

Outline of the study.

Healthy adults (18-50 years). Delivery Information Sheet and Informed Consent Form

Delivery of informed consent. Opening history. Survey frequency of food consumption.

Randomization. Delivery of CRDs

TOUR 1 DAY 1

Anthropometric measurements and clinical examination. Poll tolerance of the researcher. Blood analysis.

Collection of CRDs, feces and salive. Delivery of capsules for 4 weeks and CRDs

TOUR 2 DAY 15

Blood analysis. Clinical Examination. Survey Anthropometric measurements tolerance researcher.

Pick CRDs, feces and saliva.

TOUR 3 DAYS 43

Daily in CRD Annotation of symptoms, stool frequency and appearance. Annotation R24horas weekly test.

Anthropometric measures. Clinical Examination Survey researcher tolerance Collection of CRDs, feces and saliva.

VISIT 4 DAY 57

Daily in CRD Annotation of symptoms, stool frequency and appearance. Annotation R24horas weekly test. Daily in CRD Annotation of symptoms, stool frequency and appearance. Annotation. R24horas weekly test.

No protocol / no review CT09-November 20/39


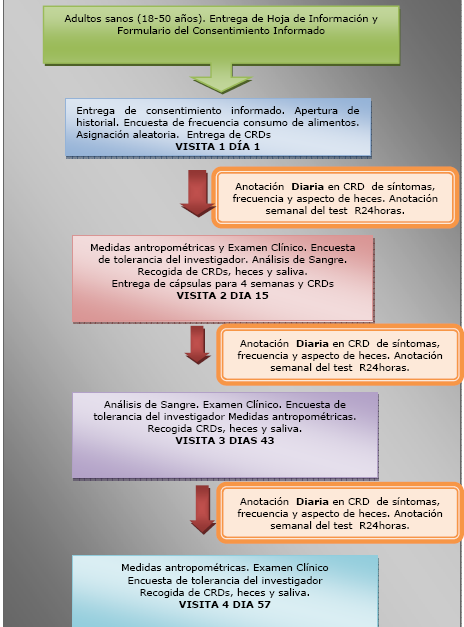


No protocol / no review CT09-November 21/39

7.7 Ethical

General considerations.

This study was performed according to the Helsinki Rules and ICH Guidelines established in terms of clinical studies in humans and in the Quality Operating Procedure Hero.

The study must be approved by the Ethical Committee of Hero Spain SA and committees University of Murcia, Hospital Universitario Virgen de las Nieves of Granada and the University of Valencia.

Information for patients and informed consent. Falls on the researcher's responsibility to give all study participants information (ANNEX II) and responsibility to voluntarily sign the form informed consent (Appendix III), before participating in this clinical trial. The investigator participants extensively explain the nature, purpose and duration planned the study. The researcher inform volunteers of the existence of a cell of attention to volunteer for any questions related to the trial.

Notice.

Treatment, communication and transfer of personal data of subjects participating in the test shall comply with the provisions of Organic Law 15/1999, of December 13, of Protection of Personal Data, and consist expressly consent reported. Likewise, participants undertake to keep confidential the information provided to them regarding the study

Liability insurance.

The Promoter of the study, Hero Spain SA has a policy of liability insurance that protects the total number of study volunteers.

No protocol / no review CT09-November 22/39

7.8. Sampling and analytical methodology.

SAMPLING

Equipment needed

All materials will be supplied for each one of the institutions participating in the project except vacuum tubes for blood collection to be provided by each one of the extraction.

• Material for extractions and blood collection tubes.

• Eppendorf type tubes for plasma aliquoted after centrifugation

• Automatic pipettes plasma aliquoted in eppendorf and pipette tips.

• Storage Boxes pipes to freeze.

• Cytochex (to preserve blood for determination of lymphocyte subsets.)

• sterile wide mouth cans for making stools.

• anerogen envelopes.

• Salivette.

Sampling

There will be fasting blood sample at the beginning and end of the intervention period. Also be taken feces and saliva samples at the beginning, at the end of the intervention period and after two weeks after finishing the operation.

Blood:

The blood sampling was carried out in four tubes as explained below (see Table takes samples):

Tube 1: 5ml EDTA tube. Steps to follow:

1st. Removal: Remove about 5 ml of blood. Check that the tube is filled completely.

2: Preparation of the aliquot with Cytochex: Immediately, you take 1 ml of blood drawn and added to a glass tube containing 1ml of Cytochex. Mix gently for about seconds, and label with a permanent marker or appropriate labels with the label that determine. The mixture should remain refrigerated until 96 hours BEFORE shipping by courier system cold. Send to the laboratory of Prof. Gaspar Ros,

No protocol / no review CT09-November 23/39

Department of Food Science and Nutrition, University of Murcia. For shipment before 96 hours, it should be consider two things

1) Tell 2 days in advance if possible, to be sent X amount of samples,

emails: esther.matencio @ hero.es and carlosgg@um.es or phone 968 89 89 00 ext 1166)

2) For the transmission itself, call the company courirer deemed by each group

properly and safely. The full mailing address is:

Technology Department, Nutrition and Food Science

Faculty of Veterinary

University of Murcia (UMU)

Espinardo

30,071 Murcia

3) The balance of the tube will be sent to the hospital laboratory for analysis of the CBC.

Tube 2: 5ml EDTA tube.

Removing approximately 5 ml of blood to obtain about 2 ml of plasma by centrifugation later. Check that the tube is completely filled.

This tube will be sent to the center laboratory relevant to the determination of the parameters Biochemical glucose, cholesterol, triglycerides, apo A-1, apo B, HDL-c, etc. ..

Tube 3: 5ml EDTA tube. Steps to follow:

Extraction 1: Extract about 5 ml blood to obtain about 2 ml plasma subsequent centrifugation.

Centrifugation and alicuotación 2nd Plasma: were centrifuged at 1000 xg for 10 min at 4 ° C, the Plasma was separated into several aliquots:

• A: To determine adipocitokinas: 0.2 ml (Send to Murcia, Institute)

• B: To determine factors of inflammation: 0.2 ml (Send at Granada University)

• C: To determine other potential biomarkers: 0.2 ml (Send to Murcia, Institute)

• D: Freeze the remaining serum in aliquots of 0.2 ml (Send to Murcia, Institute)

Tube 4: Biochemical tube without anticoagulant (with agar):

Placing 3 ml of blood and the serum separated by centrifugation. Separated serum should serve to determining IgA and other Ig if necessary.

After centrifugation at 1000 xg for 10 min and 4 ° C aliquots were made as follows:

No protocol / no review CT09-November 24/39

• E: For determination of immunoglobulins: 0. 5 ml (Shipping to Murcia, Institute)

• F: PCR determination altasensibilidad To: 0. 5 ml (Shipping to the same laboratory that performs the CBC)

• M: For metabolomics possible future analysis, tubes are placed in specially branded eppendorf: 0.2 ml (Shipping to Murcia, Institute)

NOTE: The rates remain at -80 ° C until shipment or analysis, with its case, sending samples should be maintaining the freeze aliquots in snow carbonic

SAMPLING OF LEE

The stool will be taken at the same location of the blood draw using two containers wide mouth provided in the act. Feces were collected and put directly into the jars, filling half of its capacity and will close immediately. One of the boats usher in an anaerobic bag (see Annex 7). The samples were transported to the laboratory of Analysis for processing in a time not exceeding 3 hours of sampling thereof.

Once received in the laboratory will proceed as follows:

From the sample taken from the anaerobic bag:

• For the PCR analysis, an aliquot is taken from at least 3 g in a plastic tube sterile freeze immediately at -80 ° C. Subsequently sent to the Institute of Hero Child Nutrition.

• Another aliquot of approximately 3 g of fresh stool sample is placed in a bag stomacher filter for microbiological plate count (see Annex 11)

• Another 3 g aliquot was processed as follows for FISH analysis.

Fecal suspension preparation for FISH

• Prepare fresh solution of paraformaldehyde (PFA) at 4%.

• Use 16% PFA in sealed ampoules supplied by the Granada Group.

• Take a 10 ml ampoule

• Add 13 ml of PBS 3X

No protocol / no review CT09-November 25/39

• Add 20 ul of 5M NaOH (2 g NaOH in 10 ml water destillada) freshly prepared (NaOH solution very quickly is carbonated)

• Add 15 ml of milliQ water or equivalent

• Adjust pH to 7.2 with 1N HCl

• Filter through a sterile 0.22μm pore

Note: Use preferably within 24 hours after preparation and never later than 5 days of the solution stored at +4 ° C.

Sample preparation for FISH analysis:

• Suspend 1 g of stool sample in 9 ml of PBS kept anaerobic environment in a sterile glass-stoppered 20-50 ml containing a magnetic stirrer sterile and swirl gently for a few minutes to complete homogeneity.

• transfer 6 aliquots of 0.2 ml sterile tubes epindorf 1.5 ml.

• Add 0.6 ml of 4% paraformaldehyde freshly prepared (see below).

• Mix by gentle inversion.

• Incubate overnight at 4 ° C to fix the cells.

• Store at -80 ° C until analysis (These aliquots are called PFA-stocks and are diluted 40X).

Sending samples to Granada on dry ice must be notified in advance to the Dr. Carolina Gómez Llorente e-mail: gomezll@ugr.es Mobile: 686 458 391. Be submitted to the following address:

Department of Biochemistry and Molecular Biology II

Institute of Nutrition and Food Technology

Biomedical Research Center

University of Granada

Health Campus

Armilla 18100, Granada, Spain

Preparation of samples to detect and quantitate fecal bacteria resistant to ampicillin and / or tetracycline.

No protocol / no review CT09-November 26/39

Stool samples should be fresh. By taking the samples should be stored in anaerobic atmosphere using envelope generators anaerobiosis (AnaeroGen) and should be stored at refrigeration temperatures. These analytics will be performed according to protocol analysis (Annex 10). They will be in the microbiology laboratories of three cities.

SAMPLING OF SALIVA

To avoid variations in the content of secretory IgA in the saliva, the saliva samples taken always early in the day.

Before taking samples volunteers should not eat or drink 30 minutes before.

For sampling system is used Salivette

Centrifuge samples at 3000 rpm for 10 minutes. Then deposited on tubes 10 ml pre-labeled with the subject identification code, date and the number of visits.

The supernatants are frozen at -20 ° C until analysis.

Subsequently, these samples will be sent by courier to the Institute of Child Nutrition Hero once you have collected all the samples of all volunteers in the corresponding medical services, at visit 4. The Medical Service or else the corresponding department, contact the Child Nutrition Institute Hero, via email esther.matencio @ hero.es or phone (968 89 89 00 EXT 1166) to organize the collection of samples.

LABELING AND DELIVERY OF SAMPLES

The coding of the samples will be with a letter and numbers in the following order:

Letter (hospital):

G samples taken at the Hospital Virgen de las Nieves of Granada

M samples taken at the University of Murcia

V samples taken at the University of Valencia

Patient code

Letter (center) - Code of the subject - aliquot-time

No protocol / no review CT09-November 27/39

The last five numbers of the medical history

Aliquot:

According to the sampling table

Suggested intervention time

September 15 to November 15

Sampling 1st start September 30 and 3-4days subsequent

Take 2nd ending October 30 and 3-4 days following

3rd takes two weeks to complete the operation (for faeces and saliva): 15

November 3-4 and subsequent days

Examples stopper M34141A1 and MA1; G10239B2 cap and GB2

The lettering is done with permanent marker recognized brand preferably to prevent is deleted, it is not recommended to use stickers. The samples were frozen immediately after its division into aliquots at -30 ° C. Subsequently transported in dry ice and frozen at -80 ° C until processing.

All aliquots were sent to Murcia, Granada and Valencia as specified.

TABLE. SAMPLING

Letter ul CENTER Analysis

BLOOD

Tube 1: EDTA Tube

Cytochex aliquot (1ml add cytochex) 1000 +1000 K Murcia, UMU CBC rest -Center or Hospital

extraction

Tube 2: Tube with EDTA (plasma samples)

General Biochemical analyzes (glucose, cholesterol, triglycerides ...)

Center or Hospital

extraction

Tube 3: Tube with EDTA (plasma samples)

Adipokines 200 ul

Institute of Nutrition Child Hero Other biomarkers 200, B, C, D B to Granada,University. C and D at the Institute

No protocol / no review CT09-November 28/39

Child Nutrition

Hero

Tube 4 with agar (serum samples) IgA and other immunoglobulins 500 E

Institute of Nutrition

Child Hero

Highly sensitive PCR 500 F

Hospital corresponding Metabolomics 200 M

Institute of Nutrition

Child Hero

LEE x

Samples in paraformaldehyde for FISH G Granada, University Frozen samples for quantitative PCR, SCFA, pH and fecal enzyme activity.

H Institute of Nutrition

Child Hero

Samples for antibiotic resistance R Valencia, Biopolis

Murcia, UMU, Granada, UGR

SALIVA

Samples in tubes Salivette

S Institute of Nutrition

Child Hero

ANALYTICAL METHODOLOGY

SAFETY AND TOLERANCE

• Hematologic parameters (red cells, hemoglobin, hematocrit, MCV, MCH, MCHC, leukocytes, segmented neutrophils, eosinophils, basophils, lymphocytes, platelets). Function liver (AST, ALT, GT), kidney (creatinine), others (cholesterol, HDL-cholesterol, cholesterol:

HDL-cholesterol ratio, glucose). Measured in selected hospitals and medical services. Measured at visit 2 and 3.

• Clinical Examination (Auscultamiento, blood pressure, heart rate) Measured in services selected physicians and hospitals.

No protocol / no review CT09-November 29/39

• Detection of lactic flora resistant to ampicillin and tetracycline in feces at 2, 6 and 8 weeks from the signing of informed consent (visits 2, 3 and 4). Stool samples must be fresh. By taking the samples must be stored in an atmosphere of anaerobically using generators on anaerobiosis (AnaeroGen) and must stored at refrigeration temperatures (see Annex 10)

Round 1

ALL DUE PROCESS CONDITIONS OF INFERTILITY HIGHEST

POSSIBLE.

1. Heavy 1 g of sample (if not have sufficient sample, which score collected) and dissolve with 9 ml of sterile saline (dilution 1).

2. Preparation of serial decimal dilutions in sterile saline in a range that covering a dilution estimated above and at least two lower dilutions (eg if estimated a count of 10^8^ cfu / g, at least prepare a range of -6 to -9).

Note: If you do not have a rough idea, there will be dilution from -1 to -9 in the first samples, to know the approximate range.

3. Sow on surface 100 ul of each dilution were plated in MRS-Amp, MRS-C-Amp, TSA-Amp, MRS-Tet, MRS-C-and TSA-Tet ​​Tet (recipe attached) to concentrations established (2, 4 and 8 mg / ml). Sow three plates / dilution.

4. Incubate for 30 h in a laboratory oven at 37 ° C in anaerobiosis generator (AnaeroGen, Oxoid).

Round 2

1. Homogeneity monitoring colony morphology on plates and visualization to 2-3 colonies microscope.

2. Given as the average value between replicas of that plate with a number of colonies between 30 and 300.

Calculation formula (to 1 g of sample):

cfu / g = average colonies x dilution x 10

• Tolerance of products. Through case report data researcher (ANNEX V) and notebook volunteer data collection (Appendix VI). These notebooks will be collected by the Medical Service at visit 2, 3 and 4.

No protocol / no review CT09-November 30/39

Biomarkers BACTERIAL COLONIZATION AND EFFECT PROBIOTIC

Stool samples are collected according to the protocol (Appendix 7). Once in delivery are dealt laboratory assays for bacterial count (FISH, RT-PCR), identification strains (RT-PCR), AGCC, secretory IgA in the faeces, pH and moisture.

• Detection of the strains in feces RT-PCR

Will be used a quantitative method based on chain reaction (PCR) previously using DNA extracted from stool samples. The primers used and equipment (RT-PCR) to identify the 3 strains.

• Bacterial count in feces by RT-PCR

To investigate the presence of bifidobacteria and lactobacilli in the intestine and its evolution during the study period, therefore, a quantitative method based on the chain reaction reaction (PCR) using DNA extracted from stool samples previously. The primers and equipment used (RT-PCR) to gender identification and quantification set the number of bifidobacteria and lactobacilli (Matsuki al; 2004). Be quantified lactobacilli, bifidobacteria, clostridia and Bacteroides PCR quantitative. Be held in the Laboratories of the Institute of Child Nutrition Hero.

• Bacterial count in plate Bifidobacteria, lactobacilli, anaerobic Total and colonization of strains CNCM I-4035 and CNCM I-4036.

Assays for certifying the proportion of the strains CNCM I-4035 (B.Breve) and CNCM I-4036 (L.rhamnosus) also showing that is workable. The bacterial count was held in 10 subjects in each group (5 groups). Following this protocol will be conducted in laboratories microbiological of the 3 cities involved in the trial (see Annex 11). DNA samples extracted will be sent to the Institute of Child Nutrition Hero for confirmation by RT-PCR and the University of Granada for confirmation of colonization of the strains Rhamnosus Lactobacillus CNCM I-4036 and Bifidobacterium breve CNCM I-4035 by analysis technique polymorphisms terminal restriction fragments (TRFLPs) (see Annex 12)

• Analysis of polymorphisms of terminal fragments (TRFLPS).

No protocol / no review CT09-November 31/39

The stool sampling and obtaining bacterial colonies will be in accordance with the protocol described in Annex 11.

At baseline (t0), 5 subjects were selected controls. They get 10 colonies grown on specific medium for bifidobacteria and 10 colonies grown in specific medium L. rhamnosus.

At the end of the study intervention (t1), 5 subjects were selected from groups B, C and D. Group B will be obtained 10 colonies grown in specific medium for bifidobacteria.

Group C were selected 10 colonies grown in specific medium L. rhamnosus.

Group D 20 colonies were selected, 10 for the specific medium bifidobacteria and 10 to specific means of L. rhamnosus.

From these colonies, DNA was extracted using the QIAamp DNA Stool kit (QIAGEN), according to the manufacturer's recommendations.

Once the DNA was checked spectrophotometrically its quality, and amplify the 16S rRNA gene using the universal oligos 27F and 1492 R, marked fluorescentemene to amplify the 16S rRNA gene using universal primers 27F and 1492R, fluorescently labeled at its 5 'end with 6-FAM and HEX, respectively (Sigma-Aldrich Corp., St. Louis, MO, USA). The amplifications were performed in duplicate, and will be treated as independent samples.

Table 1. Specific sequence fluorescently labeled primers.

Primer (27F) Fluorophore Color

6-FAM AGAGTTTGATCMTGGCTCAG Blue

Indirect primer (1492R) Fluorophore Color

HEX TACGGYTACCTTGTTACGACTT Green

It will remove excess dNTPs and primers and approximately 400 ng of the product Amplified be digested with restriction enzyme HhaI (New England Biolabs, Ipswich, MA). The product once dialysate is injected into a 3130XL Genetic Analyzer (Applied Biosystems,Foster City, CA), along with a molecular weight marker, also marked a fluorophore (GeneScanTM500ROXTM, Applied Biosystems, Foster City, CA), which allows analysis fragments between a size from 35 to 500 base pairs generated by the action the restriction enzyme.

No protocol / no review CT09-November 32/39

After obtaining the corresponding electroforegramas, these will be analyzed with the software GeneMapper v4.0 (Applied Biosystems Foster City, CA). The size, in basepairs, of the Restriction fragments (TRFLPs) is estimated in reference to the internal standard (50-500 bp) using the local method or the 3rd Least souther squeare. Fragments with a peak height of less than 50 GRANTED fluorescence units of analysis. Samples are analyzed in replicate, selecting only the common peaks between replicates.

These data will be analyzed using the program T-REX (Culman et al., 2009). This program allows us to analyze in a fast and robust data from the TRFLPs. Data obtained were compared with the results obtained TRFLPs of probiotic bacteria pure.

• Total number of bacteria FISH.

Bifidobacterium genus, Bacteroides group, Enterobacteriaceae, Streptococcus group, Lactobacillus group, Atopobium cluster, Clostridium coccoides group, Clostridium leptum group, Clostridium perfringens and Clostridium difficile sps sps, using specific probes labeled with fluorophores and As coupled to the flow cytometer.

• Analysis of short chain fatty acids.

Assay was performed by gas chromatography according to the method described by Girard Pipau et al. (2002). The carrier gas is helium at a flow of 20 ml / min. The air flow is 400 ml / min and the hydrogen flow is 35 ml / min. The injector temperature is 250 ° C and detector 300 ° C. The initial oven temperature is 90 ° C. The initial time is 2 minutes. The increasing temperature ranges are between 60-120 ° C. The final time is 8 minutes.

Were quantified following acids: acetic, propionic, isobutyric, butyric, isovaleric valeric isocaproic, caproic and heptanoic acid. Labs will be held at the Institute of Hero Child Nutrition.

• Analysis of enzymatic activity in faeces After homogenizing the fecal samples is determined fecal enzymatic activity by APIzym system. Be conducted in the laboratories of the Institute of Child Nutrition Hero.

No protocol / no review CT09-November 33/39

Biomarkers IMMUNOLOGIC

Measures of percentages blood lymphocyte populations, T lymphocytes (CD3 +), B lymphocytes (CD19 +) cells, T helper (CD4 +), cytotoxic T lymphocytes (CD8 +), suppressor T cells (CD4 + CD25 +), NK cells (CD56 +) and monocytes (CD14 +). Be measured by flow cytometry in blood samples collected in vacutainers containing EDTA. Were measured at the University of Murcia, Department of Food Science.

Plasma cytokines (TNF-α, IL-12, IL-10, TGF-β, IL-4, IL-12) measured at the University of Granada, Department of Biochemistry and Molecular Biology II, using the multiplex system. Will determine the concentration of IgA in feces, saliva and plasma by enzyme immunoassay (ELISA) for the homogenized samples in PBS buffer at 10% (w / v) according to the method of Bakker-Zierikzee AM, 2006. In the laboratories of the Institute of Nutrition Child Hero.

Analysis of C-reactive protein in plasma (hsCRP)

Anthropometric Measures

The data for weight, height, body mass index corpuscular will be collected during the visits by the service physician in the frequency established in clinical trial design. Conducting these measures in the Medical Service of the University of Murcia, Valencia and University Hospital Virgen de las Nieves in Granada

9. ANALYTICAL DATA COLLECTION

The study data were collected in electronic format (Microsoft Excel) and treatment Statistical data was performed using the SPSS 18.0 statistical package. Data are expressed as the mean ± standard deviation. A p <0.05 was considered significant.

The normality of continuous variables will be compared using the Shapiro-Wilk test. When normally distributed variables comparison between groups thereof, will be out using a Student t test for unpaired data. When continuous variables normally distributed to the product comparison test does not apply parametric (Mann-Whitney). The sex distribution in the two study groups analyzed by Chi-square test. To study the data, is an analysis of variance (ANOVA) and multifactorial using confidence intervals of 95%.

No protocol / no review CT09-November 34/39

10. ACTIVITY OR TASK

Group 1: Literature review on the aspects of the project.

Research groups involved: HERO.

Activity 1: Review of the literature on clinical studies in adults tolerance and security probiotics functionality

Activity Dates: Start: January 2011. End: March 2011.

Activity 2: Creating the trial protocol. Made by Hero Spain, SA

Activity Dates: Start: February 2011. End: April 2011.

Group 2: Preliminary Guidelines for the conduct of the study.

Research groups involved (with initials): HERO, UGR, UMU, Biopolis

Activity 1: Application and approval of the study by the Ethics Committee for Presentation of the study "in vivo" the Ethics Committee of each of the centers involved in the present study for approval. Meeting all requirements for your approval.

Date of Activity: Start: March-2011. End: May-2011

Activity 2: Leaves of informed consent and preparation of documentation for the Data collected during the study.

Preparation of informed consent forms, patient information sheets, daily intake and tolerance, notebook and data collection procedures for archiving and use the document cited.

Date of activity: Start: February-2011. End: May-2011

No protocol / no review CT09-November 35/39

Activity 3: Manufacture of capsules to probiotics.

Manufacture of the products under study and microbiological checks necessary prior to consumption by the volunteers.

Date of activity: Start: August-2011. End: August -2011

Group 3: Development of analytical methodology.

Research groups involved (with initials): HERO, UMU, UGR

Activity 1: Data collection intake and tolerance.

Activity conducted in three centers (UMU, UGR and Biopolis) during scheduled visits.

Date of activity: Start: September 2011. End: December 2011.

Activity 2: Anthropometric measurements, physical examination and blood count.

Activity carried out by the medical service of the three universities (UMU, UGR and Biopolis)

performing at scheduled visits.

Date of activity: Start: September 2011. End: December 2011.

Activity 3: Measurements of pH, SCFA, fecal IgA and IgA in saliva.

Activity carried out at the Institute of Child Nutrition (HERO).

Date of activity: Start: September 2011. End: December 2011.

Activity 4: Bacterial count by RT-PCR

Activity carried out at the Institute of Child Nutrition (HERO).

Activity 5: Identification of strains in feces.

Activity carried out at the Institute of Child Nutrition (HERO).

Date of activity: Start: September 2011. End: December 2011.

Activity 6: FISH in feces.

Activity carried out at the University of Granada (UGR).

Date of activity: Start: November 2011. End: December 2011.

Activity 7: Microbiological testing by bacterial culture.

No protocol / no review CT09-November 36/39

Activity carried out at the University of Granada (UGR), Biopolis, Hero.

Date of activity: Start: November 2011. End: December 2011.

Activity 8: Confirmation of colonization by RT-PCR.

Activity carried out by the Institute of Child Nutrition Hero

Date of activity: Start: November 2011. End: December 2011

Activity 9: Confirmation of colonization polymorphism analysis of the fragments terminal restriction.

Activity carried out at the University of Granada (UGR).

Date of activity: Start: November 2011. End: December 2011

Activity 10: lymphocyte count.

Activity carried out at the University of Murcia (UMU).

Date of activity: Start: November 2011. End: December 2011.

Activity 11: Measurements of plasma cytokines.

Activity carried out at the University of Granada (UGr).

Date of activity: Start: November 2011. End: December 2011.

Activity 12: Measures of ampicillin and penicillin resistance in lactic flora Activity held in BIOPOLIS.

Date of activity: Start: November 2011. End: December 2011.

Group 4. Conclusions and final report.

Research groups involved (with initials): HERO

Activity 1: Statistical treatment of data, discussion of results, conclusions.

Date of activity: Start: July 2011. End: December 2011.

Activity 2: Final Report

Date of activity: Start: July 2011. End: December 2011.

Activity 3: Preparing the possible publication of results in scientific journals highest impact.

No protocol / no review CT09-November 37/39

11. MILESTONES

MILESTONE 1: Application of the study "in vivo". Date: April -2011

MILESTONE 2: Completion of the trial design "in vivo": Date: December - 2011

MILESTONE 3: Statistical analysis of the results. Date: December 2011

MILESTONE 4: Conclusions and final report. Date: December 2011

MILESTONE 5: For publication of results in scientific journals. Date: February 2012

No protocol / no review CT09-November 38/39

12. SCHEME AND WORK PLAN.


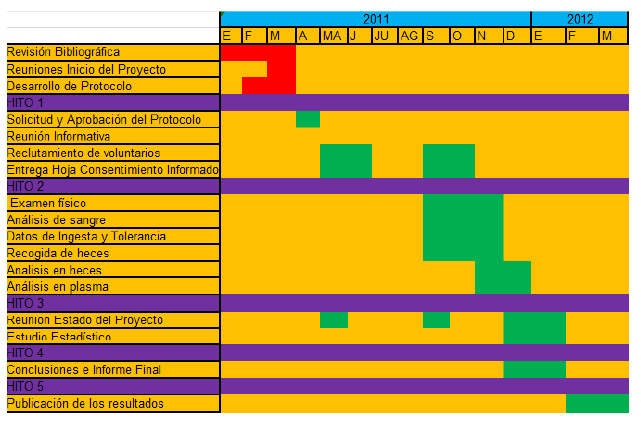


13. CONTRIBUTION OF THE PROJECT A HERO GROUP STRATEGIC PURPOSES

The completion of this clinical trial and publication of the results will be part of Hero Group knowhow. Hero Group may offer the three strains from other companies interested in using the same for use in food supplements, pharmaceutical products, functional foods for adults.

14. CONTINGENCIES AND RISKS ANTICIPATED.

There is no risk to the health of the participants in the study as a result of the intake of capsules containing the probiotic strains Hero property Spain, SA, as it These strains have been evaluated toxicologically according to the report of FAO / WHO 2002. Furthermore it has been shown by the absence of genomic sequencing plasmids containing genes for antibiotic resistance as recommended EFSA scientific proposals in 2008.

Not foresee any risk during and after the period of intake. Intake of probiotics, rarely may cause the onset of gastrointestinal symptoms (loose stools, stool hard, vomiting, nausea, abdominal pain, flatulence, belching).

No protocol / no review CT09-November 39/39

15. ECONOMIC REPORT.

Euros Budget Concept Contract Services, UMU Medical Service, population analysis

lymphocyte, CONTRACT

UGR FISH, cytokines and medical service CONTRACT

Biopolis production strains, recruitment, medical service, primers, testing antibiotic resistance in fecal CONTRACT

Hero Insurance Institute, Material sampling, PCR assays, AGCC, humidity, pH, IgA, saliva and feces.

16. ANNEXES

Annexes from 0 to 12
