# Supplementary material for: Safety and Immunomodulatory Effects of Three Probiotic Strains Isolated from the Feces of Breast-Fed Infants in Healthy Adults: SETOPROB Study
Source: PLoS One. 2013 Oct 28;8(10):e78111. doi: 10.1371/journal.pone.0078111 (PMC3810271; doi:10.1371/journal.pone.0078111)
Supplement: Table S1 — Probes used in fluorescence in situ hybridization (FISH). (DOCX) [file pone.0078111.s003.docx]

**TABLE S1.** Probes used in fluorescence *in situ* hybridization (FISH)

| Probes | Sequence from 5’ to 3’ end | Targeted groups | Reference |
| --- | --- | --- | --- |
| Bif164 | CATCCGGCATTACCACCC | *Bifidobacterium* genus | 18 |
| Bac303 | CCAATGTGGGGACCTT | *Bacteroides* group | 19 |
| Enter1432 | CTTTTGCAACCCACT | *Enterobacteriacea* | 20 |
| Str493 | GTTAGCCGTCCCTTTCTGG | *Streptococcus* group | 21 |
| Lab158 | GGTATTAGCAYCTGTTTCCA | *Lactobacillus group* | 22 |
| Ato291 | GGTCGGTCTCTCAACCC | *Atopobium* cluster | 23 |
| Erec482 | GCTTCTTAGTCARGTACCG | *Clostridium coccoides- Eubacterium rectale* group | 21 |
| Clep866 | GGTGGATWACTTATTGTG | *Clostridium leptum* group | 24 |
| Cpef191 | GCTCCTTTGGTTGAATGATG | *Clostridium perfringens* | 25 |
| Cdif198 | TCCATCCTGTACTGGCTCACC | *Clostridium difficile* | 25 |
