# Supplementary material for: Safety and Immunomodulatory Effects of Three Probiotic Strains Isolated from the Feces of Breast-Fed Infants in Healthy Adults: SETOPROB Study
Source: PLoS One. 2013 Oct 28;8(10):e78111. doi: 10.1371/journal.pone.0078111 (PMC3810271; doi:10.1371/journal.pone.0078111)
Supplement: Table S2 — Primer sequences used in real-time PCR. (DOCX) [file pone.0078111.s004.docx]

**TABLE S2.** Primer sequences used in real-time PCR

| **Microbial target** | Strain used for standard curve | **Sequence 5´🡪 3¨** |
| --- | --- | --- |
|  |  |  |
| *Bifidobacterium* spp. | *B. longum* CECT 4503 | CTCCTGGAAACGGGTGG (Forward) |
|  |  | GGTGTTCTTCCCGATATCTACA (Reverse) |
| *B. longum* | B longum CECT 4503 | TTCCAGTTGATCGCATGGTCT (Forward) |
|  |  | GGCTACCCGTCGAAGCCACG (Reverse) |
| *B. breve* | *B breve* CECT 4839 | CCGGATGCTCCATCACAC (Forward) |
|  |  | ACAAAGTGCCTTGCTCCCT (Reverse) |
| *B. dentium* | *B dentium* CECT 687 | ATCCCGGGGGTTCGCCT (Forward) |
|  |  | GAAGGGCTTGCTCCCGA (Reverse) |
| *B. bifidum* | *B bifidum* DSM 20456 | CCACATGATCGCATGTGATTG (Forward) |
|  |  | CCGAAGGCTTGCTCCCAAA (Reverse) |
| *B. catenulatum* | *B. catenulatum* DSM 16992 | GCCGGATGCTCCGACTCCT (Forward) |
|  |  | ACCCGAAGGCTTGCTCCCGAT (Reverse) |
| *B. adolescentes* | *B adolescentes* CECT 5781 | CTCCAGTTGGATGCATGTC (Forward) |
|  |  | CGAAGGCTTGCTCCCAGT (Reverse) |
| *Lactobacillus* | *L. paracasei* CNCM-I-4034 | TGGATGCCTTGGCACTAGGA (Forward) |
|  |  | AAATCTCCGGATCAAAGCTTAC (Reverse) |
| *Bacteroides fragilis* | *Bacteroides fragilis* DSM 2151 | GAGAGGAAGGTCCCCCAC (Forward) |
|  |  | CGCTACTTGGCTGGTTCAG (Reverse) |
| *Clostridium difficile* | *Clostridium difficile* CECT 531 | TTGAGCGATTTACTTCGGTAAAGA (Forward) |
|  |  | TGTACTGGCTCACCTTTGATATTCA (Reverse) |
| *Lactobacillus rhamnosus* CNCM I-4036 | *Lactobacillus rhamnosus* CNCM I-4036 | TGGCACTCACTGCAATTCGT (Forward) |
|  |  | GATGCTTTGGCGTTGGTGTA (Reverse) |
