# Supplementary material for: Safety and Immunomodulatory Effects of Three Probiotic Strains Isolated from the Feces of Breast-Fed Infants in Healthy Adults: SETOPROB Study
Source: PLoS One. 2013 Oct 28;8(10):e78111. doi: 10.1371/journal.pone.0078111 (PMC3810271; doi:10.1371/journal.pone.0078111)
Supplement: Table S3 — Frequency and consistency of the feces. (DOCX) [file pone.0078111.s005.docx]

**TABLE S3.** Frequency and consistency of the feces

|  | Probiotic groups (n=80) | | |  | Placebo group (n=20) | | |
| --- | --- | --- | --- | --- | --- | --- | --- |
|  | t_1_ | t_2_ | t_3_ |  | t_1_ | t_2_ | t_3_ |
| **Stool consistency** |  |  |  |  |  |  |  |
| Watery | 3 | 2 | 3 |  | 2 | 2 | 3 |
| Normal | 74 | 75 | 73 |  | 17 | 16 | 16 |
| Hard | 3 | 3 | 4 |  | 1 | 2 | 1 |
| **Frequency** |  |  |  |  |  |  |  |
| # of daily deposition | 1.2 ± 0.6 | 1.5 ± 0.4 | 1.4 ± 0.2 |  | 1.1 ± 0.3 | 1.2 ± 0.6 | 1.2 ± 0.4 |

Stool consistency values are based on the volunteers’ responses to a questionnaire. Frequency of deposition is expressed as means ± SEM.
