# Supplementary material for: Safety and Immunomodulatory Effects of Three Probiotic Strains Isolated from the Feces of Breast-Fed Infants in Healthy Adults: SETOPROB Study
Source: PLoS One. 2013 Oct 28;8(10):e78111. doi: 10.1371/journal.pone.0078111 (PMC3810271; doi:10.1371/journal.pone.0078111)
Supplement: Table S4 — Percentage of antimicrobial resistance populations. (DOCX) [file pone.0078111.s006.docx]

**TABLE S4.** Percentage of antimicrobial resistance populations

| Culture media | Capsule | Time (t) | |
| --- | --- | --- | --- |
|  |  | t_1_ | t_2_ |
| TSA+Ampicillin (2 µg/mL) | Placebo | 1.45 ± 0.99 | 3.02 ± 0.43 |
|  | *L. rhamnosus* | 4.11 ± 0.85 | 4.08 ± 0.04 |
|  | *B. breve* | 0.50 ± 0.13 | 0.34 ± 0.18 |
|  | *B. breve* plus *L. rhamnosus* | 3.36 ± 0.89 | 3.07 ± 0.33 |
|  | *L. paracasei* | 5.79 ± 0.51 | 6.07 ± 0.55 |
| TSA+Ampicillin (4 µg/mL) | Placebo | 0.15 ± 0.08 | 0.13 ± 0.01 |
|  | *L. rhamnosus* | 0.05 ± 0.02 | 0.01 ± 0.01 |
|  | *B. breve* | 0.06 ± 0.01 | 0.02 ± 0.02 |
|  | *B. breve* plus *L. rhamnosus* | 0.22 ± 0.12 | 0.22 ± 0.01 |
|  | *L. paracasei* | 0.54 ± 0.14 | 0.37 ± 0.09 |
| MRS+Ampicillin (2 µg/mL) | Placebo | 8.30 ± 2.60 | 7.96 ± 1.67 |
|  | *L. rhamnosus* | 5.32 ± 3.81 | 3.65 ± 0.83 |
|  | *B. breve* | 2.24 ± 1.42 | 1.36 ± 1.13 |
|  | *B. breve* plus *L. rhamnosus* | 5.12 ± 1.90 | 6.64 ± 2.85 |
|  | *L. paracasei* | 5.67 ± 1.07 | 4.62 ± 1.89 |
| MRS+Ampicillin (4 µg/mL) | Placebo | 4.65 ± 3.27 | 5.05 ± 1.10 |
|  | *L. rhamnosus* | 3.34 ± 1.63 | 2.55 ± 0.71 |
|  | *B. breve* | 2.30 ± 1.67 | 2.29 ± 0.60 |
|  | *B. breve* plus *L. rhamnosus* | 5.45± 2.21 | 4.28 ± 1.56 |
|  | *L. paracasei* | 5.87 ± 0.47 | 4.26 ± 1.82 |
| MRSC+Ampicillin (2 µg/mL) | Placebo | 0.050 ± 0.030 | 0.019 ± 0.017 |
|  | *L. rhamnosus* | 0.012 ± 0.010 | 0.012 ± 0.001 |
|  | *B. breve* | 0.002 ± 0.003 | 0.002 ± 0.003 |
|  | *B. breve* plus *L. rhamnosus* | 0.018 ± 0.012 | 0.014 ± 0.002 |
|  | *L. paracasei* | 0.009 ± 0.002 | 0.004 ± 0.003 |
| MRSC+Ampicillin (4 µg/mL) | Placebo | 0.043 ± 0.033 | 0.033 ± 0.028 |
|  | *L. rhamnosus* | 0.009 ± 0.001 | 0.010 ± 0.001 |
|  | *B. breve* | 0.002 ± 0.003 | 0.001 ± 0.001 |
|  | *B. breve* plus *L. rhamnosus* | 0.010 ± 0.006 | 0.013 ± 0.002 |
|  | *L. paracasei* | 0.005 ± 0.001 | 0.003 ± 0.001 |
| TSA+Tetracycline (4 µg/mL) | Placebo | 0.28 ± 0.08 | 0.16 ± 0.05 |
|  | *L. rhamnosus* | 0.15 ± 0.07 | 0.19 ± 0.02 |
|  | *B. breve* | 0.02 ± 0.01 | 0.01 ± 0.01 |
|  | *B. breve* plus *L. rhamnosus* | 0.09 ± 0.04 | 0.03 ± 0.01 |
|  | *L. paracasei* | 0.16 ± 0.05 | 0.20 ± 0.02 |
| TSA+Tetracycline (8 µg/mL) | Placebo | 0.19 ± 0.07 | 0.24 ± 0.04 |
|  | *L. rhamnosus* | 0.07 ± 0.01 | 0.05 ± 0.01 |
|  | *B. breve* | 0.02 ± 0.01 | 0.01 ± 0.01 |
|  | *B. breve* plus *L. rhamnosus* | 0.08 ± 0.02 | 0.04 ± 0.01 |
|  | *L. paracasei* | 0.09 ± 0.02 | 0.12 ± 0.01 |
| MRS+Tetracycline (4 µg/mL) | Placebo | 1.95 ± 0.68 | 2.94 ± 0.72 |
|  | *L. rhamnosus* | 2.81 ± 2.12 | 1.57 ± 0.91 |
|  | *B. breve* | 4.01 ± 2.37 | 2.51 ± 1.81 |
|  | *B. breve* plus *L. rhamnosus* | 8.79 ± 3.14 | 6.31 ± 1.49 |
|  | *L. paracasei* | 2.79 ± 1.70 | 3.27 ± 1.18 |
| MRS+Tetracycline (8 µg/mL) | Placebo | 1.83 ± 0.99 | 0.85 ± 0.25 |
|  | *L. rhamnosus* | 2.66 ± 2.38 | 0.93 ± 1.10 |
|  | *B. breve* | 1.97 ± 1.72 | 1.79 ± 0.25 |
|  | *B. breve* plus *L. rhamnosus* | 7.89 ± 2.36 | 6.45 ± 1.51 |
|  | *L. paracasei* | 2.15 ± 1.12 | 2.21 ± 0.74 |
| MRSC+Tetracycline (4 µg/mL) | Placebo | 0.06 ± 0.04 | 0.03 ± 0.02 |
|  | *L. rhamnosus* | 0.05 ± 0.02 | 0.01 ± 0.01 |
|  | *B. breve* | 0.002 ± 0.001 | 0.001 ± 0.001 |
|  | *B. breve* plus *L. rhamnosus* | 0.024 ± 0.0.22 | 0.014 ± 0.000 |
|  | *L. paracasei* | 0.006 ± 0.001 | 0.003 ± 0.002 |
| MRSC+Tetracycline (8 µg/mL) | Placebo | 0.053 ± 0.029 | 0.027 ± 0.015 |
|  | *L. rhamnosus* | 0.015 ± 0.013 | 0.012 ± 0.002 |
|  | *B. breve* | 0.002 ± 0.004 | 0.002 ± 0.002 |
|  | *B. breve* plus *L. rhamnosus* | 0.018 ± 0.013 | 0.014 ± 0.002 |
|  | *L. paracasei* | 0.001 ± 0.001 | 0.001 ± 0.001 |

Values are means ± SEM, in percentages of resistant bacteria. n=3 per group. Means within a group without a common letter differ significantly. *P*<0.05. t_1_, first washout; t_2_, intervention. MRS, Man-Rogosa-Sharpe; MRSC, Man-Rogosa-Sharpe with cysteine; TSA, Tryptone soy agar.
